# Supplementary material for: Estimating the glutamate transporter surface density in distinct sub-cellular compartments of mouse hippocampal astrocytes
Source: PLoS Comput Biol. 2022 Feb 4;18(2):e1009845. doi: 10.1371/journal.pcbi.1009845 (PMC8849624; doi:10.1371/journal.pcbi.1009845)
Supplement: S1 Appendix — This file contains the mathematical details of for estimating the maximal density of glutamate transporters in a circle, sphere and cylinder. (PDF) [file pcbi.1009845.s001.pdf]

S1 Appendix

The maximal density of glutamate transporters in a 2D circle

Our knowledge of the structure of eukaryotic glutamate transporters is largely based on the crystal structure of the outward-facing [1], inward-facing [2], open states [3], Na^+:substrate binding [4] and trimer assembly [5] of Glt_{Ph}, a glutamate transporter homolog from the prokaryote *Pyrococcus horikoshii.* Glt_{Ph} has $\sim 37\%$ (high) aminoacid identity with human GLT-1. For this reason, its structural features are currently thought to apply to other eukaryotic glutamate transporters, which have not yet been crystallized. The general consensus is that all glutamate transporters assemble as trimers, with each monomer functioning independently from the others [5]. The trimeric assembly delimits a bowl-shaped concave aqueous basin that prevents glutamate molecules unbinding from the transporters from being lost to extracellular diffusion [6] and contains molecular determinants that are involved in Cl^- channel activation of Glt_{Ph} [3]. In a 3D space, each trimer is analogous to a triangular prism. We used this information to determine: *(i)* which fraction of the surface area of different portions of the astrocytic membrane is occupied by glutamate transporters; *(ii)* how their surface density compares with the maximal density of transporters that can be reached in each compartments. Answering this questions requires the use of a geometrical approach, which we implemented starting from simplified 2D and 3D geometries. For example, we can use a circle as a 2D approximation of the astrocyte soma and the cross-section of its branches, and a rectangle with a base $\omega = 8$ and height $h = 6.5$ to represent each transporter trimer each trimer as a rectangle (**S1A-B Fig**). This approximation is based on s simplified description of the crystal structure of Glt_{Ph}, a prokaryotic homolog of glutamate transporters from *Pyrococcus horikoshii* [1].

In a circle delimited by an ideal $\psi = 3$ nm thick lipid bilayer [7], the distance from the center to the outer layer of the plasma membrane is defined as the outer radius r_1, whereas r_0 represents the distance from the center to the inner layer of the membrane, where $r_0 = r_1 - \psi$. The outer radius of the circle r_1 was allowed to vary, accounting for variability in the size of the soma and of the branch diameters. According to crystal structure analysis of the inward- and outward-facing configurations of Glt_{Ph}, the transmembrane-spanning portion of each trimer lies approximately half way through the plasma membrane [1, 2, 8]. However, there are conformational changes that occur as glutamate transporters transition between the outward- and inward-facing configuration, which allow the transporter to protrude towards the cytoplasm ($h_{in} = 3.5$ nm) or towards the extracellular space ($h_{in} = 0$ nm) [2, 5, 8–11]. For this reason, in our analysis, we include the results obtained for $h_{in} = 0$ (transporters protruding towards the extracellular space), 1.75 (transporters located half-way through the plasma membrane) and 3.5 nm (transporters protruding towards the cytoplasm). For our further computations, we also used the distance from the outside surface of the soma to the inward bottom of the trimer h_{out}, defined as follows:

$$h_{out} = h_{in} + \psi \tag{S1}$$

Therefore, h_{out} could vary between $\psi = 3$ nm (when $h_{in} = 0$) and $h_{out} = 6.5$ nm (when $h_{in} = 3.5$ nm). Having defined these parameters, we proceeded to estimate the maximum number of transporter trimers that can be fitted in the circle (n_{circle}), as well as their maximal linear density (σ_{circle}) and the maximal fraction of perimeter

occupancy (ϕ_{circle}; **S2 Fig**). The corresponding values for the transporter monomers was estimated by multiplying these values by three.

The maximum number of transporter trimers that can occupy a circle n_{circle} depends on: *(i)* the circumference of the circle (i.e., on r_1); *(ii)* the transporter trimer width ω; and *(iii)* the height of the transporter trimer protruding in the cytoplasm (h_{in}). For a fixed cytoplasmic protrusion h_{in} and when $r_1 > h_{out}$ (so that the circle can accommodate at least two trimers), the number of trimers can be maximized by placing them in a radially symmetric fashion around the circle of radius r_1, such that their inner widths delimits a regular polygon concentric with the center of the circle. Then n_{circle} is precisely the number of sides of this polygon. To estimate n_{circle}, we additionally labelled the distance r_{-1} from the center to the corners of the polygon, and the distance l from the center to the sides of the polygon (**S2A Fig**). By applying Pythagoras' rule to the triangles delimited by $(r_{-1}, l, \omega/2)$ and $(r_1, l + h_{out}, \omega/2)$, we obtained:

$$r_{-1}^2 = l^2 + \left(\frac{\omega}{2}\right)^2 \tag{S2}$$

and

$$r_1^2 = (l + h_{out})^2 + \left(\frac{\omega}{2}\right)^2 \tag{S3}$$

By subtracting **Eq (S2)** from **Eq (S3)**, we obtained:

$$r_1^2 - r_{-1}^2 = 2lh_{out} + h_{out}^2 \tag{S4}$$

We expressed $l = r_{-1}\cos(\theta)$ and $\omega = 2r_{-1}\sin\theta$, where the angle $\theta = \dfrac{\pi}{n_{circle}}$ can vary in the range $[0, \pi/2]$ (**S2A Fig**). As a result, **Eq (S4)** can be re-written as:

$$r_{-1}^2 + 2r_{-1}h_{out}\cos\theta + h_{out}^2 - r_1^2 = 0 \tag{S5}$$

By eliminating $r_{-1} = \omega/(2\sin\theta)$, **Eq (S5)** can also be re-written as:

$$\frac{\omega^2}{4\sin^2\theta} + h_{out}\omega\cot\theta - r_1^2 + h_{out}^2 = \frac{\omega^2}{4}[\cot^2\theta + 1] + h_{out}\omega\cot\theta - r_1^2 + h_{out}^2 = 0$$

This led to the quadratic equation in $\xi = \cot\theta$:

$$\frac{\omega^2}{4}\xi^2 + h_{out}\omega\xi + \frac{\omega^2}{4} - r_1^2 + h_{out}^2 = 0$$

By solving for $\xi = \cot\theta > 0$, we obtained:

$$\cot\theta = \cot\left(\frac{\pi}{n_{circle}}\right) = \frac{-2h_{out} + \sqrt{4r_1^2 - \omega^2}}{\omega} \tag{S6}$$

Based on **Eq (S6)**, the maximum number of transporter trimers of width ω and cytoplasmic protrusion height h_{in} that can occupy a circle of radius r_1 is:

$$n_{circle} = \frac{\pi}{\cot^{-1}\left(-2h_{out}/\omega + \sqrt{4r_1^2/\omega^2 - 1}\right)} \tag{S7}$$

Our computation, and subsequently **Eq (S7)**, holds true if $r_1 > h$ and $r_1 > \omega/2$ (to allow space for at least two trimer insertions into the astrocyte cytoplasm). These conditions were both satisfied experimentally, given that the radius of the finest tip of the terminal branches of astrocytes were 10-120 nm (**Table 3** in the manuscript). As expected, n_{circle} increased as r_1 increased (i.e., bigger circles contained more

transporters), and the dependence was almost indistinguishable from linear within the conditions for r_1 (**S2B-C Fig**). Increasing h_{in} values produced no changes in the graph, because any steric effect due to possible collisions between the cytoplasmic tails of adjacent transporters is negligible when $r_1 > h$ and $r_1 > \omega/2$ (**S2B-C Fig**). The maximum linear density of transporter trimers σ_{circle}, was calculated by dividing n_{circle} by the circumference length:

$$\sigma_{circle} = \frac{n_{circle}}{2\pi r_1} \tag{S8}$$

Here, the transporter density $3\sigma_{circle}$ approaches an asymptotic value of $1/\omega \sim 375$ μm^{-1}, independently of the value of h_{in}, and reaches 99% of it when $r_1 > 17$ nm (**S2D Fig**). The fraction of the circle circumference occupied by the transporters, Φ_{circle}, can be calculated as the ratio between the length of the circumference occupied by the trimers n_{circle} and that of the entire circumference. Here, the circular length occupied by the trimers can be calculated as $\hat{\omega} = 2\theta r_1 = 2r_1 \sin^{-1}\left(\frac{w}{2r_1}\right)$, and the maximum portion of the circle occupied by them is given by:

$$\Phi_{circle} = \frac{n_{circle}\hat{\omega}}{2\pi r_1} = \frac{n_{circle}\sin^{-1}(\omega/2r_1)}{\pi} = \frac{\sin^{-1}(\omega/2r_1)}{\cot^{-1}\left(-2h_{out}/\omega + \sqrt{4r_1^2/\omega^2 - 1}\right)} \tag{S9}$$

The relationship approached the asymptotic value 1, for all values of h_{in} (**S2E Fig**). This asymptotic value was reached more slowly as h_{in} increased (i.e., as the transporter trimers protruded more towards the astrocyte cytoplasm). For $h_{in} = 0$, 1.75, and 3.5 nm, 99% of the asymptotic value was reached when $r_1 = 300$, 475 and 650 nm, respectively. Note that the relationship between Φ_{circle} and r_1 was less steep than the relationship between σ_{sphere} and r_1. This was due to the fact that the dependence of the circle perimeter on r_1 grew more slowly than that of the perimeter length occupied by the monomers as r_1 increased. Together, the results of this simple 2D model indicate that crowding effects do not limit the local surface expression of glutamate transporters in large sub-cellular compartments like the soma, but can do this in small astrocytic processes, depending on the expression of other molecules.

The maximal density of glutamate transporters in a 3D sphere

A similar conceptual strategy to the one described for the 2D circle can be applied to estimate the glutamate transporter surface density in 3D, using a spherical representation of the astrocyte soma (**S1D Fig**). As in the 2D case, the geometry that maximizes the number of transporters is obtained when the cytoplasmic portions of the trimers touch each other, delimiting the triangular faces of a regular polyhedron with side length ω.

For simplicity, we used the same notation system described in the previous section, where r_1 is the external radius of the sphere, $r_0 = r_1 - \psi$ is the slightly smaller radius of the cytoplasmic portion of the sphere (accounting for the membrane thickness ψ), and r_{-1} is the radius of the sphere that circumscribes the polyhedron described by the cytoplasmic portions of the trimers. Consistent with our previous notation, we called h_{in} the depth of the cytoplasmic protrusion of the trimer, and $h_{out} = h_{in} + \psi$ the insertion depth measured from the outer portion of the plasma membrane. The radius r_{-1} was obtained as a function of r_1, ω and h_{out}. If we call l the distance from the center of the sphere to the cytoplasmic portion of each trimer, we obtain:

$$l = \sqrt{r_{-1}^2 - \omega^2/3}$$

following that:

$$l + h_{out} = \sqrt{r_{-1}^2 - \omega^2/3} + h_{out} = \sqrt{r_1^2 - \omega^2/3}$$

Solving for r_{-1}, we obtain:

$$r_{-1} = \sqrt{r_1^2 + h_{out}^2 - 2h_{out}\sqrt{r_1^2 - \omega^2/3}}$$

We estimated the number n_{sphere} of triangular prisms (i.e., the transporter trimers) distributed along the outer surface of the sphere (**S1F Fig**). Each pair of vertices of each triangle (i.e., the base of the triangular prisms) defines a great circle on the sphere (that is a circle centered at the center of the sphere). Therefore, each triangle defined three great circles (**S1C Fig**). If one calls A^{Δ_s} the area of the spherical triangle delimited by these three great circles, then:

$$n_{\text{sphere}} = \frac{4\pi r_{-1}^2}{A^{\Delta_s}} \tag{S10}$$

The area A^{Δ_s} was computed in the Methods section using a strategy for overlapping spherical lunes, as:

$$A^{\Delta_s} = r_{-1}^2 \left[3\cos^{-1}\left(\frac{2r_{-1}^2 - \omega^2}{4r_{-1}^2 - \omega^2}\right) - \pi\right] \tag{S11}$$

Hence the maximum number of transporter trimers that can be placed in a sphere can be approximated by dividing the surface are of a sphere of radius r_1 by A^{Δ_s}:

$$n_{\text{sphere}} = \frac{4\pi}{3\cos^{-1}\left(\frac{2r_1^2 + 2h_{out}^2 - 4h_{out}\sqrt{r_1^2 - \omega^2/3} - \omega^2}{4r_1^2 + 4h_{out}^2 - 8h_{out}\sqrt{r_1^2 - \omega^2/3} - \omega^2}\right) - \pi} \tag{S12}$$

The maximum surface density of the trimers can then be expressed by:

$$\sigma_{\text{sphere}} = \frac{n_{\text{sphere}}}{4\pi r_1^2} \tag{S13}$$

The number and surface density of transporter monomers can be obtained by multiplying n_{sphere} and σ_{sphere} by three.

The lateral sides of each transporter trimer cut a spherical triangle of area $A^{\Delta}_{\text{sphere}}$ out of the plasma membrane (i.e., from the sphere of radius r_1). The area of this spherical triangle was calculated as a double iterated integral of the spherical surface of radius r_1 over the triangular domain of side length w centered at the origin. In the Methods section, we used polar coordinates to rewrite this area in terms of a double integral, and compute it as:

$$A^{\Delta}_{\text{sphere}}(r_1) = 2\pi r_1^2 - 3r_1^2\left[2\tan^{-1}\left(\frac{3r_1}{\sqrt{3r_1^2 - \omega^2}}\right) - \frac{\omega}{\sqrt{3}r_1}\sin^{-1}\left(\frac{\sqrt{3}\omega}{\sqrt{12r_1^2 - \omega^2}}\right)\right] \tag{S14}$$

Therefore, the maximum fraction of the sphere surface occupied by transporter trimers is:

$$\Phi_{\text{sphere}} = \frac{n_{\text{sphere}} \cdot A^{\Delta}_{\text{sphere}}(r_1)}{4\pi r_1^2} \tag{S15}$$

where $A^{\Delta}_{\text{sphere}}$ is provided by **Eq (S14)**. The results of this analysis, summarized in (**S3 Fig**), show that the number of transporters on the surface of a sphere increases with the sphere radius r_1. In this case, however, the relationship increases faster than if it was linear (cf. **S3C Fig** and **S2C Fig**). For a small radius ($r_1 < 0.1$ nm), the transporter density increased steeply as r_1 got larger, but this behavior eventually tapered off as r_1 increased, eventually approaching an asymptotic value of $10.8 \cdot 10^4$ monomers μm^{-2}. As the transporters protruded more towards the astrocyte cytoplasm, the asymptotic value was approached more slowly for increasing values of r_1. Accordingly, 99% of the asymptotic value was reached at $r_1 = 488$, 768 and 1057 nm, respectively as h_{in} increased from 0 to 1.75 and 3.5 nm. The relationship between n_{sphere} and r_1 was less steep than the one between n_{circle} and r_1, which we described for the 2D case (cf. **S3 Fig** and **S2D Fig**). This held true when calculating the proportion of a sphere of increasing radius that can be occupied by the transporters (cf. **S3E Fig** and **S2E Fig**). In this case, 99% of the sphere surface could be occupied by transporter monomers at $r_1 = 601$, 949, 1,298 nm, respectively, as h_{in} increased from 0 to 1.75 and 3.5 nm. Together, these results show that changes in the conformational state of glutamate transporters, leading to changes in the insertion level in the plasma membrane, are unlikely to create steric hindrance limits in large sub-cellular compartments like the soma, but can do so in small bulbous protrusions that astrocytes form, especially if their radius is only a few tens or hundreds nm.

The maximal density of glutamate transporters in a 3D cylinder

Each astrocyte branch, or its portions, can be roughly approximated by a cylinder (**S1E Fig**). Given that the diameter of a branch (i.e., a cylinder) is typically larger than the summed height of two trimers 2χ, the configuration that maximizes trimer packing along horizontal rings is the one shown schematically in **S4A-B Fig**. Here, a cylinder is formed by a stack of rings, and each ring has height $H = \sqrt{3}\omega/2$, which corresponds to the height of the triangular base of a trimer.

If one considers a cross section of a cylinder that is perpendicular to its axis, it is possible to recover the circular geometry of the 2D model described previously, where each rectangle corresponds to a pair of trimers. This observation allowed us to calculate the maximum number of trimers that can be assembled along this cylindrical ring of radius r_{cyl} as twice the number of rectangles that can fit around the circular cross section of the cylinder, as calculated for a circle:

$$n_{\text{ring}} = \frac{2\pi}{\cot^{-1}\left(-2h_{out}/\omega + \sqrt{4r_{cyl}^2/\omega^2 - 1}\right)} \tag{S16}$$

where $h_{out} = h_{in} + \psi$. A cylinder of length L consists of $L/H = 2L/\sqrt{3}\omega$ stacked rings. Therefore, the total number of trimers along a cylinder of length L and radius $r_{cyl} > h_{out}$ is given by:

$$n_{cyl}(r_{cyl}, L) = \frac{2\pi}{\cot^{-1}\left(-2h_{out}/\omega + \sqrt{(2r_{cyl})^2/\omega^2 - 1}\right)} \cdot \frac{2L}{\sqrt{3}\omega} \tag{S17}$$

For a given value of L, the trimer number n_{cyl} increases approximately linearly **S4C Fig**. The maximum surface density of trimers in a cylinder of length L can be expressed as:

$$\sigma_{cyl} = \frac{n_{cyl}}{2\pi r_{cyl} L} \tag{S18}$$

The maximum monomer density is obtained by multiplying σ_{cyl} by three (**S4D Fig**). Here, the maximum transporter surface density $3 \cdot \sigma_{cyl}$ approaches an asymptotic value of 10.82e4 μm^{-2}. Accordingly, 99% of this asymptotic value is reached at $r_{cyl} = 282$ nm, 445 nm, and 608 nm, respectively, as h_{in} increased from 0 to 1.75 and 3.5 nm (**S4D Fig**). Values significantly lower than the asymptotic value could be detected in small cylinders with $r_{cyl} < 0.1$ μm, meaning that a crowding effect can limit the local surface density of the transporters in very small astrocytic processes whose geometry can be approximated to that of cylinders. The area $A^{\Delta}_{cyl}(r_{cyl})$ that each trimer occupies on the surface of a cylindrical extension of radius r_{cyl} was calculated in the Methods section as a double integral:

$$A^{\Delta}_{cyl}(r_{cyl}) = \sqrt{3}\omega r_{cyl} \sin^{-1}\left(\frac{\omega}{2r_{cyl}}\right) + \sqrt{3} r_{cyl}\sqrt{4r_{cyl}^2 - \omega^2} - 2\sqrt{3} r_{cyl}^2 \tag{S19}$$

From this formula, we calculated the proportion of the cylindrical surface area occupied by trimers as:

$$\Phi_{cyl} = \frac{n_{cyl}(r_{cyl}, L) \cdot A^{\Delta}_{cyl}(r_{cyl})}{2\pi r_{cyl} L} \tag{S20}$$

where $n_{cyl}(r_{cyl}, L)$ and $A^{\Delta}_{cyl}(r_{cyl})$ are given by **Eq (S17)** and **Eq (S19)**, respectively. The protrusion of the trimers towards the lumen of the cylinder introduced a crowding effect that prevented them from occupying the entire lateral surface of the cylinder. Accordingly, 99% of the asymptotic value 1 was reached at $r_{cyl} = 302$, 476, and 651 nm, respectively, as h_{in} increased from 0 to 1.75 and 3.5 nm (**S4E Fig**). This means that astrocytic processes with terminal processes that are only 18-238 nm wide (see **Table 3** in the manuscript) may have an upper limit to the number of transporters they express due to their small size.

Since our model is based on assembling multiple cylindrical segments into the 3D simulations of the astrocyte branching tree, we need to consider the transitional geometry between these cylinders at branching points, as well as the effects of the potential constraints of this geometry on the membrane distribution of transporter trimers. At a typical branching point (illustrated in **S5A Fig**), the parent branch (with diameter D) generates a daughter branch (with diameter d, at an angle θ with the original direction) and continues along the same direction, with a smaller diameter Δ (determined by the 3/2 rule). The geometry at the branching point is an interpolation of all these structures, forming approximately the lateral surface of a truncated cone of height h_{bp} (shown in blue in **S5A Fig**), with an opening for the base of the daughter branch. For our model computations, we considered the exact volume of this transitional branch point portion, as

$$V_{\text{bp}} = \frac{\pi h_{bp}}{12}(D^2 + \Delta^2 + D\Delta)$$

and we approximated the surface area (for which there is no precise elementary formula) as the lateral area of a cylinder with height $h_{bp} = d \tan\theta$, and diameter Δ (shown by the dotted lines in the figure):

$$A_{bp} = \pi h_{bp} \Delta$$

This cylindrical approximation accounts for the opening in the membrane where the daughter branch departs from the parent branch, and is also used to calculate the number of trimers that can be placed on the surface of the blue truncated cone. We

used two simplifications to deal with the effect of the complex geometry at the branching point on the local transporter distribution. *First*, we assumed that trimers are placed optimally on the surface of the parent branch, which will prevent other transporters from occupying the initial portion of the membrane of the daughter branch (shaded red region on the figure). This region (red shaded area in **S5A Fig**) is a slant cylinder with its highest length $x = h_{out}/\sin\theta + h_{out}/\tan\theta$ and smallest length $y = h_{out}\sin\theta$. Its effect is almost equivalent to assuming that a portion of length $(x+y)/2$ of the daughter branch is devoid of trimers. *Second*, a portion of length $z = h_{out}/\tan\theta$ on the parent branch after the branching point is also devoid of trimers due to a "shadow" effect generated by the generation of the daughter branch.

Another exception to the assumption of cylindrical shapes arises at the tips of terminal branches, which are better approximated by the shape of a small hemisphere. Since the diameters of the terminal branches are typically very small, the additional curvature of the tips makes them another location where spacial constraints are likely to lead to geometric hindrance for trimer distribution. To estimate the size of this effect with our simulations, we considered the small hemispherical tips as a separate model cell compartment (see below). The theoretical transporter number, density and fraction of the membrane occupied by transporters can then be compared between this compartment and other parts of the cell, to infer the effect of the additional geometric constrains present at the tip. As a basic approximation (illustrated in **S5 Fig**, and detailed mathematically in **Eq S21-S26**), notice that these measures tightly agree for a hemispherical tip and for a cylindrical tip of the same height (assuming no transporters are placed on the end of the cylindrical portion).

$$n_{htip}(r) = \frac{2\pi}{3\cos^{-1}\left(\frac{2r^2 + 2h_{out}^2 - 4h_{out}\sqrt{r^2-\omega^2/3} - \omega^2}{4r^2 + 4h_{out}^2 - 8h_{out}\sqrt{r^2-\omega^2/3} - \omega^2}\right) - \pi} \tag{S21}$$

$$n_{ctip}(r) = \frac{2\pi}{\cot^{-1}\left(-2h_{out}/\omega + \sqrt{4r^2/\omega^2 - 1}\right)} \cdot \frac{2r}{\sqrt{3}\omega} \tag{S22}$$

$$\sigma_{htip} = \frac{n_{htip}}{4\pi r^2} \tag{S23}$$

$$\sigma_{ctip} = \frac{n_{ctip}}{2\pi r^2} \tag{S24}$$

$$\Phi_{htip} = \frac{n_{htip} A_{sphere}^{\Delta}(r)}{4\pi r^2} \tag{S25}$$

$$\Phi_{ctip} = \frac{n_{ctip} A_{cyl}^{\Delta}(r)}{2\pi r^2} \tag{S26}$$

References

1. Dinesh Yernool, Olga Boudker, Yan Jin, and Eric Gouaux. Structure of a glutamate transporter homologue from Pyrococcus horikoshii. *Nature*, 431(7010):811, 2004.

2. Nicolas Reyes, Christopher Ginter, and Olga Boudker. Transport mechanism of a bacterial homologue of glutamate transporters. *Nature*, 462(7275):880, 2009.

3. Ichia Chen, Shashank Pant, Qianyi Wu, Rosemary J Cater, Meghna Sobti, Robert J Vandenberg, Alastair G Stewart, Emad Tajkhorshid, Josep Font, and Renae M Ryan. Glutamate transporters have a chloride channel with two hydrophobic gates. *Nature*, 591(7849):327–331, 2021.

4. C Alleva, Kirill Kovalev, R Astashkin, MI Berndt, C Baeken, T Balandin, Valentin Gordeliy, Ch Fahlke, and J-P Machtens. Na+-dependent gate dynamics and electrostatic attraction ensure substrate coupling in glutamate transporters. *Science Advances*, 6(47):eaba9854, 2020.

5. Grégory Verdon and Olga Boudker. Crystal structure of an asymmetric trimer of a bacterial glutamate transporter homolog. *Nature Structural & Molecular Biology*, 19(3):355, 2012.

6. Greg P Leary, David C Holley, Emily F Stone, Brent R Lyda, Leonid V Kalachev, and Michael P Kavanaugh. The central cavity in trimeric glutamate transporters restricts ligand diffusion. *Proceedings of the National Academy of Sciences*, 108(36):14980–14985, 2011.

7. Gerrit Van Meer, Dennis R Voelker, and Gerald W Feigenson. Membrane lipids: where they are and how they behave. *Nature Reviews Molecular Cell Biology*, 9(2):112, 2008.

8. Olga Boudker, Renae M Ryan, Dinesh Yernool, Keiko Shimamoto, and Eric Gouaux. Coupling substrate and ion binding to extracellular gate of a sodium-dependent aspartate transporter. *Nature*, 445(7126):387, 2007.

9. Nurunisa Akyuz, Roger B Altman, Scott C Blanchard, and Olga Boudker. Transport dynamics in a glutamate transporter homologue. *Nature*, 502(7469):114, 2013.

10. Maarten Groeneveld and Dirk-Jan Slotboom. Na^+:aspartate coupling stoichiometry in the glutamate transporter homologue Glt_{Ph}. *Biochemistry*, 49(17):3511–3513, 2010.

11. Grégory Verdon, SeCheol Oh, Ryan N Serio, and Olga Boudker. Coupled ion binding and structural transitions along the transport cycle of glutamate transporters. *eLife*, 3, 2014.
