# Supplementary figures and images for: Estimating the glutamate transporter surface density in distinct sub-cellular compartments of mouse hippocampal astrocytes
Source: PLoS Comput Biol. 2022 Feb 4;18(2):e1009845. doi: 10.1371/journal.pcbi.1009845 (PMC8849624; doi:10.1371/journal.pcbi.1009845)

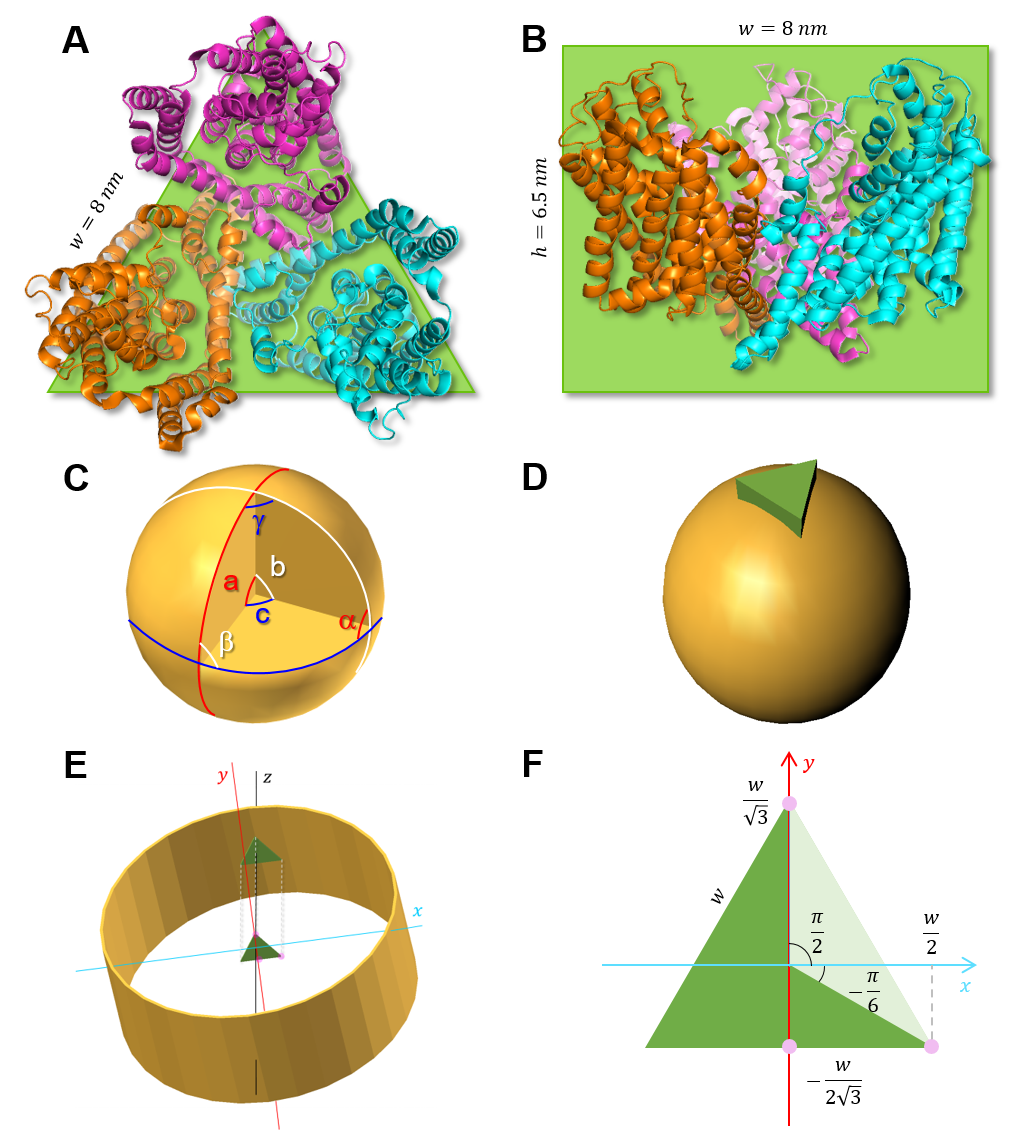

Supplement: S1 Fig — A. Extracellular view of the ribbon representation of a GltPh trimer, with protomers shown in orange, magenta and cyan. The green triangle with side length w = 8 nm is used to approximate the cross section of the trimer. B. View of the trimer parallel to the membrane. The green rectangle with h = 6.5 nm height is used to approximate the side view of the trimer. C. Spherical triangle (formed by three intersecting lunes) with spherical angles α, β and γ, corresponding to angles at the center of the sphere called a, b and c, respectively. D. Schematic representation of a trimer (green triangular prism) protruding through the plasma membrane of a spherical compartment. E. Schematic representation of a transporter protruding inside a cylindrical compartment; the bottom of the trimer and its projection onto the x, y Cartesian coordinate plane are shown as green triangles. F. Projection of the trimer onto the coordinate plane, used as the domain for computing the double integrals for AsphereΔ and AcylΔ. (TIF) [file pcbi.1009845.s002.tif]

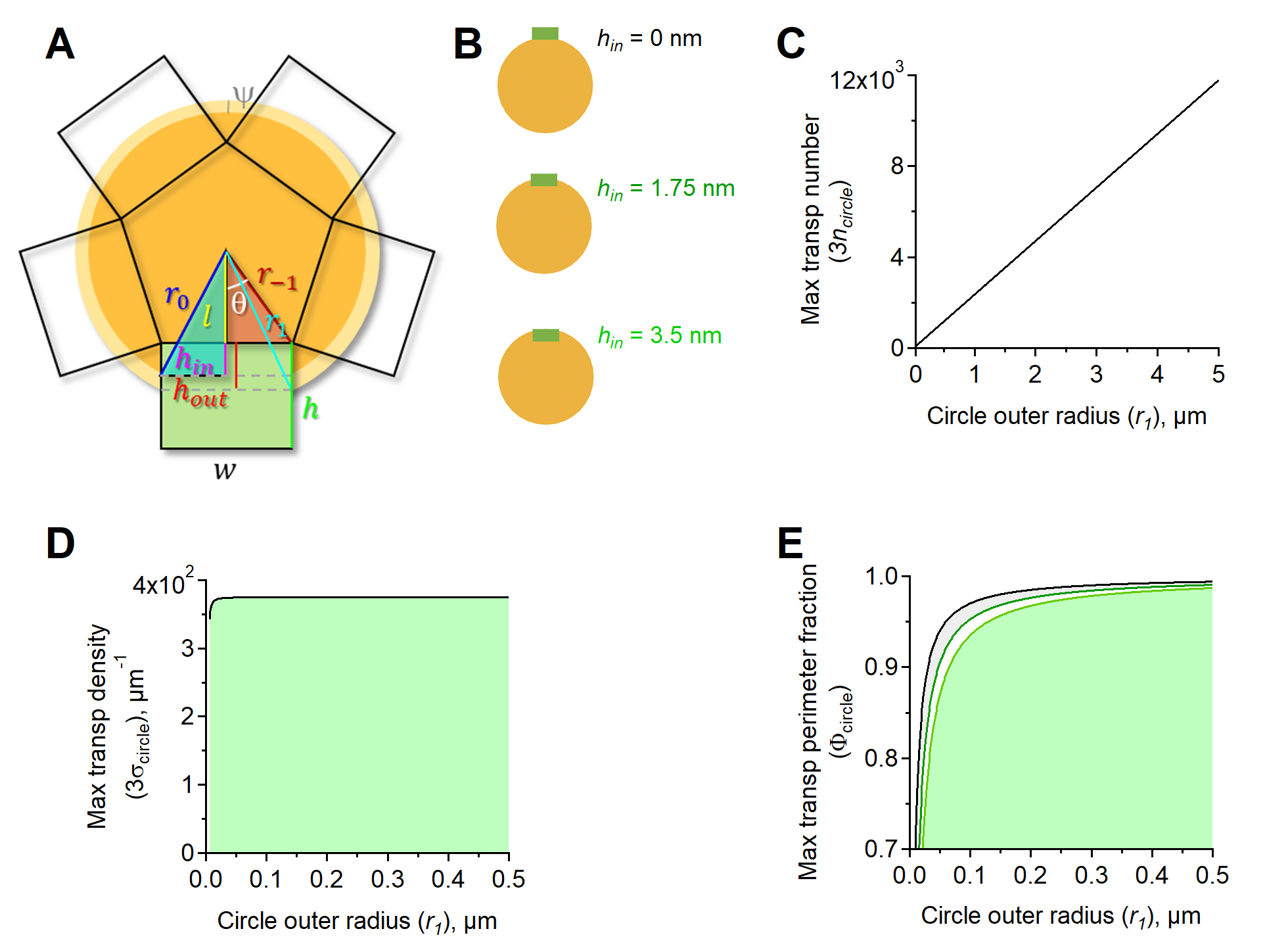

Supplement: S2 Fig — A. Each transporter trimer had a portion of length (hin) that could protrude inside the circle from the cytoplasmic layer of the plasma membrane. The side length of the trimers was ω = 8 nm. This spatial arrangement provided a framework to estimate the maximum number of transporter trimers and monomers that can reside in the perimeter of a circle (ncircle) of radius r1. B. Schematic representation of circles with transporter trimers. The glutamate transporter trimers were represented as rectangles with 8 nm side length and 6.5 nm height. The example at the top refers to trimers that do not protrude into the circle. In the example in the middle, the trimers were located half way through the plasma membrane and protruded in the circle by 1.75 nm. In the case described at the bottom, the trimers protruded all the way through the membrane, for 3.5 nm. C. Estimates of the maximum number of transporter molecules (3ncircle) that can be positioned along the circumference of circles of different radius r1. We examined the three different cases described in (A), where hin = 0 nm (black), hin = 1.75 nm (dark green) and hin = 3.5 nm (light green). D. Estimates of the 2D glutamate transporter monomer density for each value of hin. E. Proportion of the circle perimeter occupied by transporter monomers for increasing values of the circle radius. An asymptotic value of 1 was reached at varying rates depending on the value of hin. (TIF) [file pcbi.1009845.s003.tif]

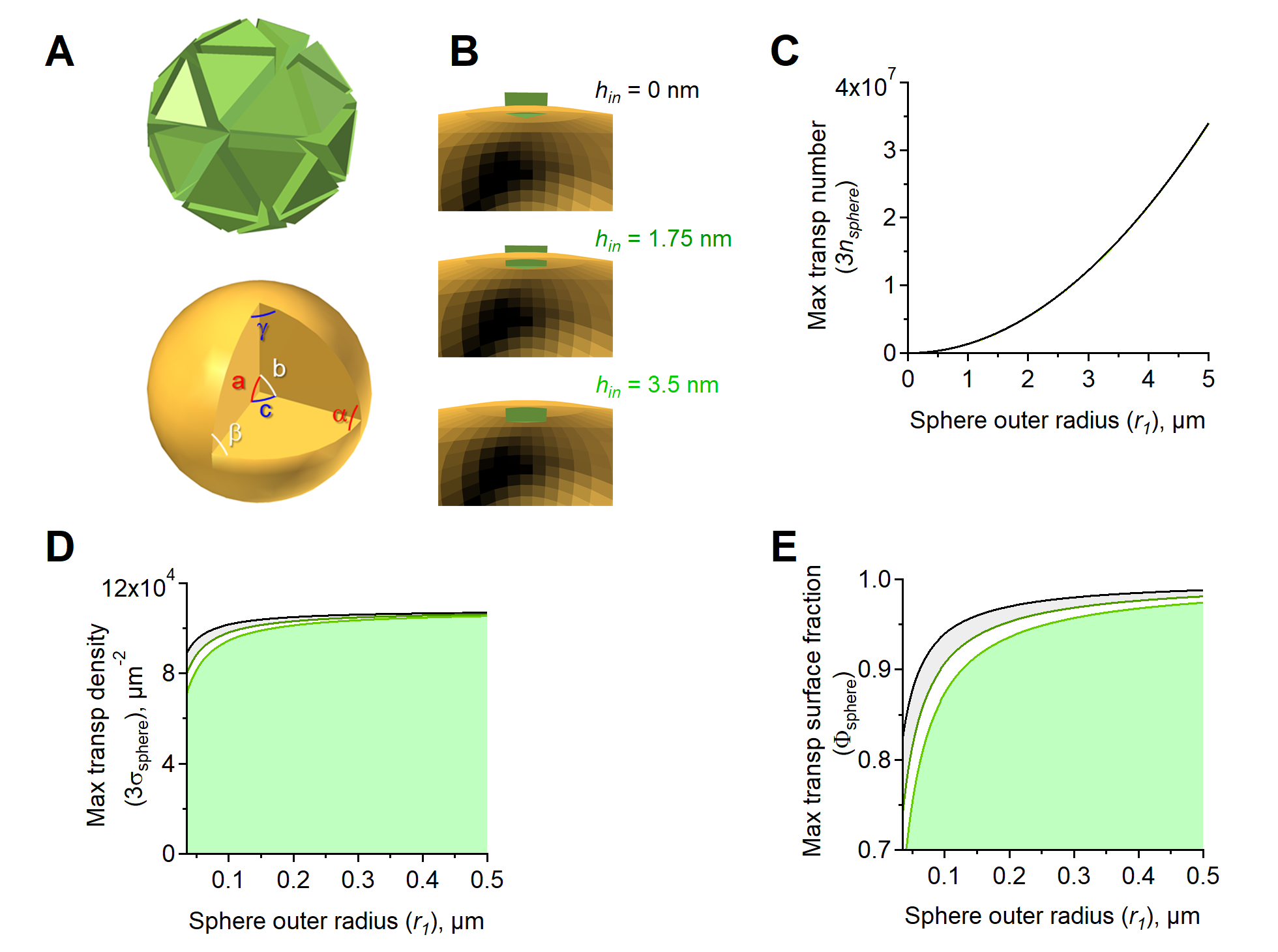

Supplement: S3 Fig — A. Top, Spatial distribution of transporter trimers in a sphere. Bottom, A spherical triangle is a figure formed on the surface of a sphere by three great circular arcs intersecting pairwise in three vertices. The spherical triangle is the spherical analog of the planar triangle. The figures shows a spherical triangle with angles a, b, c, equivalent to the angles α, β, γ. B. Schematic representation of spheres used as 3D representations of the astrocyte soma. Glutamate transporter trimers are represented as triangular prisms with a base of 8 nm side length and 6.5 nm height. The example at the top refers to trimers that do not protrude into the sphere. In the example in the middle, the trimers are located half way through the plasma membrane and protrude in the sphere by 1.75 nm. In the case described at the bottom, the trimers protrude all the way through the membrane, for 3.5 nm. C. Estimates of the maximum number of transporter trimers ns phere that can be positioned along the surface of spheres of different radius r1. We examined the three different cases described in (B), where hin = 0 nm (black), hin = 1.75 nm (dark green) and hin = 3.5 nm (light green). D. Estimates of the 3D transporter trimer surface density for each value of hin. E. Fraction of the surface area of the sphere that can be occupied by transporter trimers. (TIF) [file pcbi.1009845.s004.tif]

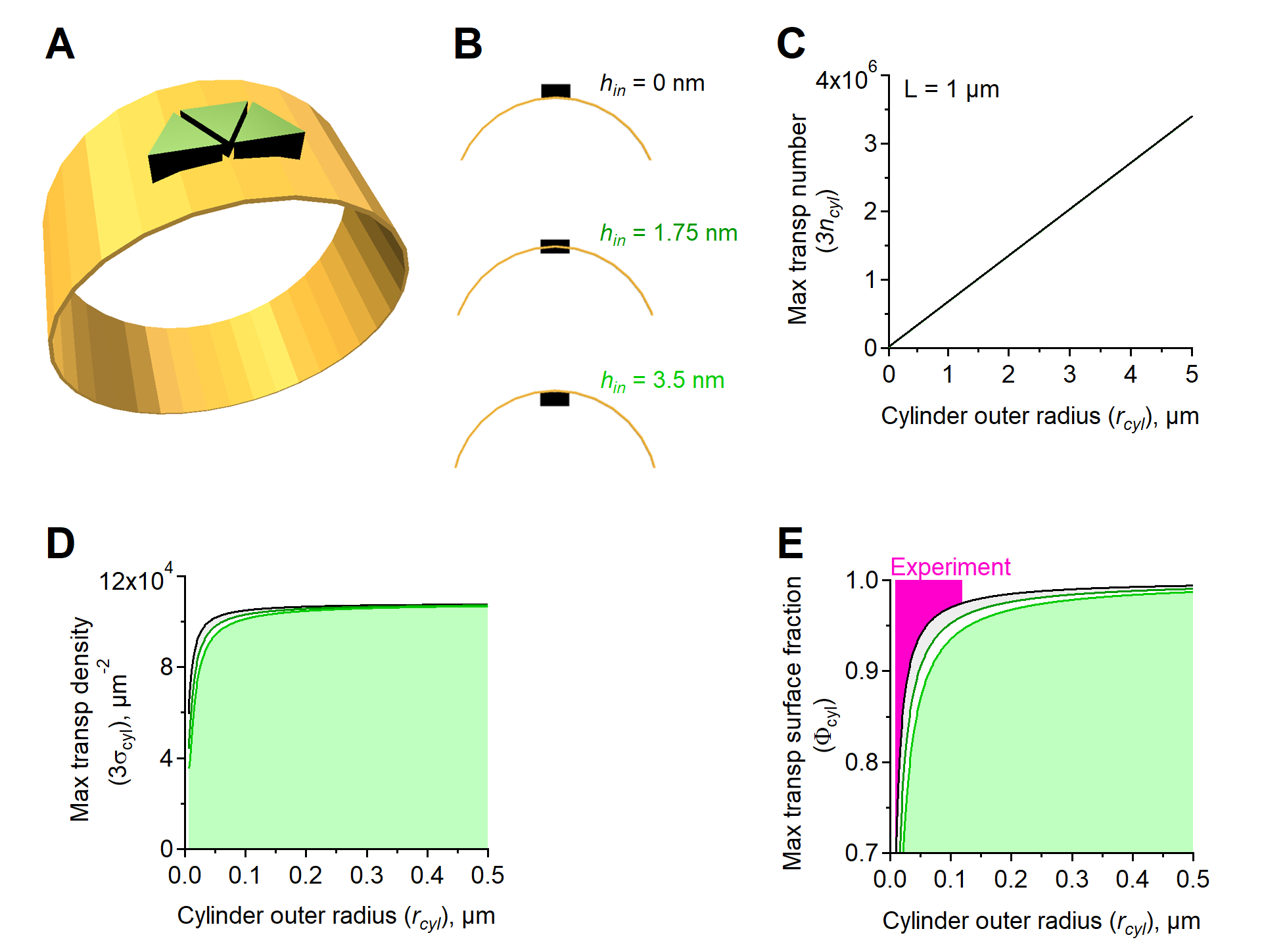

Supplement: S4 Fig — A. Schematic representation of trimer arrangement around the lateral surface of a cylinder. For clarity, we only show three adjacent trimers, but in reality they keep covering the entire lateral surface of the cylinder using the same pattern. B. Schematic representation of cylinders used as 3D representations of astrocytic branches. Glutamate transporter trimers are represented as triangular prisms with a base of 8 nm side length and 6.5 nm height. The example at the top refers to trimers that do not protrude in the lumen of the cylinder. In the example in the middle, the trimers are located half way through the plasma membrane and protrude inside the cylinder by 1.75 nm. In the case described at the bottom, the trimers protrude all the way through the membrane, for 3.5 nm. C. Estimates of the maximum trimer number ncyl that can be positioned along the lateral surface of cylinders of different radius rcyl (the height of the cylinder in this case is set to L = 1 μm. We examined the three different cases described in (B), where hin = 0 (black), hin = 1.75 (dark green) and hin = 3.5 nm (light green). D. Estimates of trimer surface density for each value of hin. E. Estimates of the fraction of the lateral surface of the cylinder that can be occupied by trimers for each value of hin. The area shaded in magenta represents the range of experimental measures for tip diameters. (TIF) [file pcbi.1009845.s005.tif]

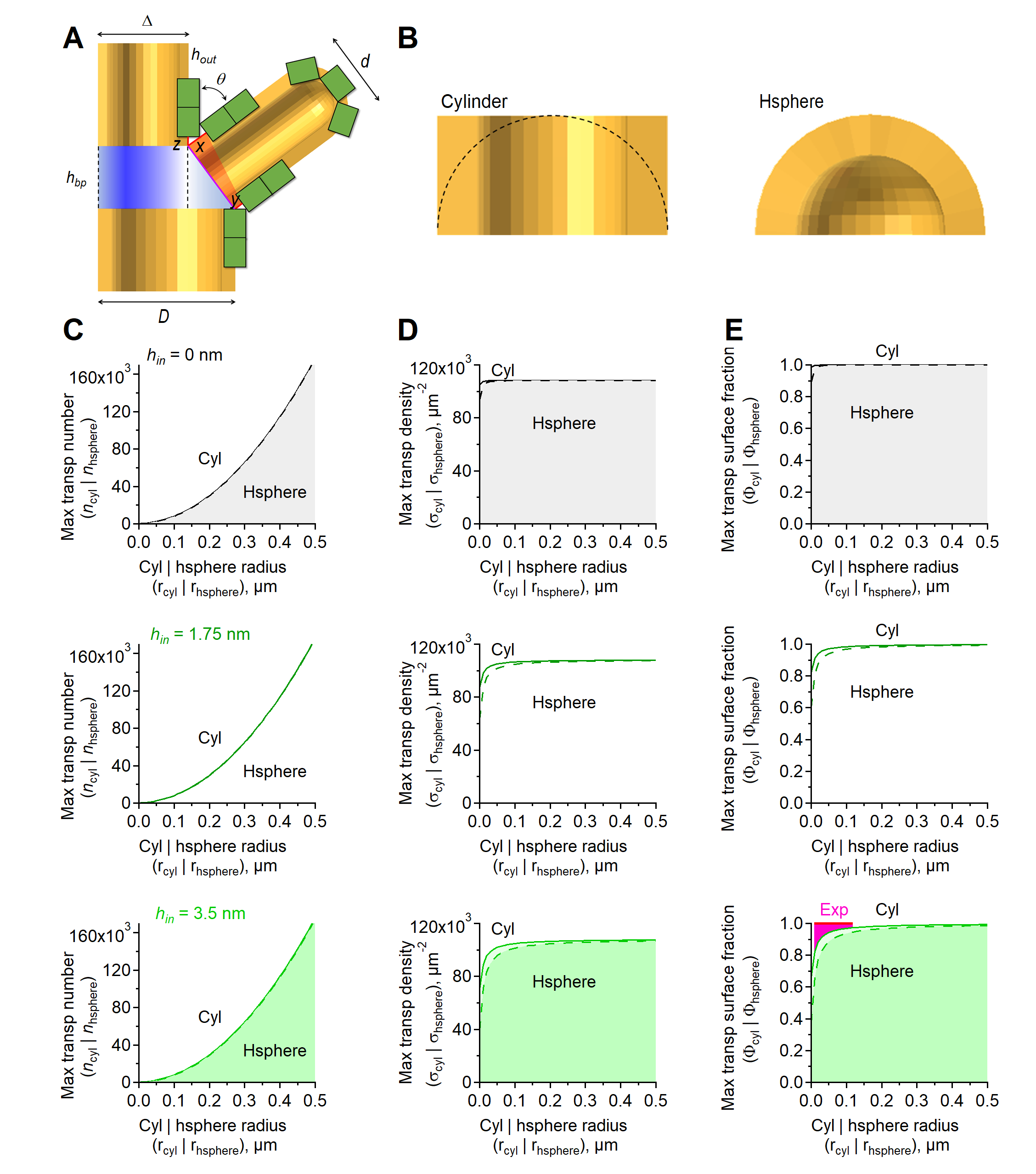

Supplement: S5 Fig — A. The illustration shows in cross section the astrocyte geometry at a branch point and at a branch tip. Each transporter trimer is represented as a green rectangle of height h. In this specific illustration, the trimer is protruding out of the plasma membrane by hout, but our calculations were performed for hin = 0–3.5 nm. The branching points and branch tip are regions that display a crowdedness effect, which reduces the local density of transporter trimers (as further explained in the text). B. Close-up view of the tip of an astrocyte process ending either as a cylinder (left) or as a hemisphere (right). C. Estimates of the maximum trimer number that can be placed in a cylinder with height equals to the radius (solid line) or in a hemisphere (dashed line), for hin = 0 nm (top), hin = 1.75 nm (middle) and hin = 3.5 nm (bottom). D. As in C, for trimer density. E. As in C, for the portion of the plasma membrane occupied by transporter trimers. (TIF) [file pcbi.1009845.s006.tif]

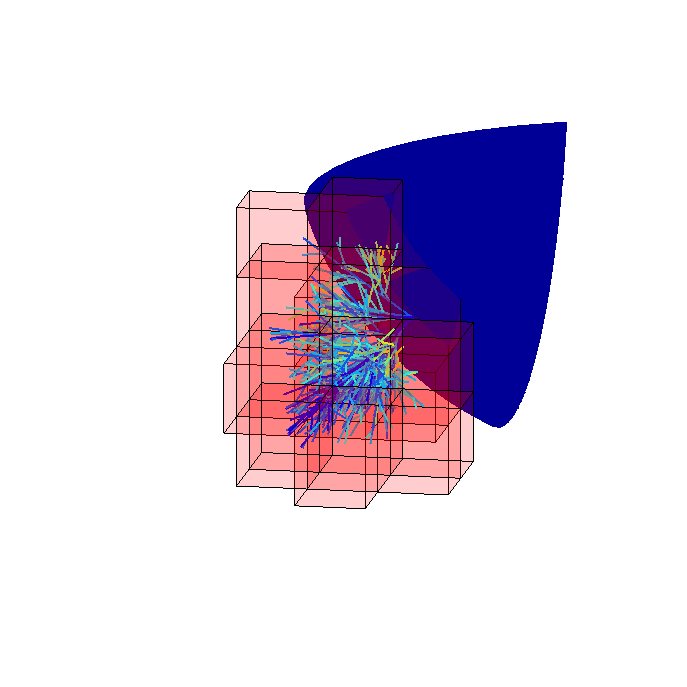

Supplement: S6 Fig — Simulated surrounding volume for an example cell with 3 primary branches, using a mesh of cubes with side length of 2 μm (shown in red). The blue wedge represents a portion of the astrocyte soma. The astrocyte processes departing from the soma are color coded based on their branch level. (TIF) [file pcbi.1009845.s007.tif]
