# Supplementary material for: Estimating the glutamate transporter surface density in distinct sub-cellular compartments of mouse hippocampal astrocytes
Source: PLoS Comput Biol. 2022 Feb 4;18(2):e1009845. doi: 10.1371/journal.pcbi.1009845 (PMC8849624; doi:10.1371/journal.pcbi.1009845)
Supplement: Attachment — Submitted filename: Plos_Comp_Bio_reviewers_letter.pdf [file pcbi.1009845.s008.pdf]

Dear Dr. Berry,

We would like to thank the reviewers for their thoughtful and thorough evaluation of this manuscripts. We believe that addressing their concerns allowed us to significantly improve the clarity and quality of the work presented here. The main points addressed in this revised version of the manuscript are listed below. *First*, we provide new experimental estimates of the glutamate transporter surface density, obtained from a wide age range in mice (2 weeks - 21 months). *Second*, we obtained analogous measures from 7-8 week old rats, which allowed us to reproduce previous results from the literature [1], and gave us confidence about the reliability of the novel estimates that we obtained from mice. *Third*, we refined our geometrical models to include a more accurate representation of the astrocyte morphology, which includes fine terminal processes, leaves, and more accurate considerations about the effect of branching angles on the local transporter density. *Fourth*, we also repeated our 3D Monte Carlo simulations to obtain a finer measure of the glutamate concentration inside the cleft, at the edges of the synaptic cleft, and in the extracellular space, with varying locations of the glutamate release sites. *Fifth*, we now discuss more recent literature on astrocyte structure and glutamate transporter distribution, which was not done in sufficient depth in the first version of this work. As a result, the manuscript now includes numerous new findings. This work provides an evaluation of how these estimates change with mouse age, which had also not been previously investigated quantitatively over such a wide age range as the one investigated here. This is the first study to provide quantitative numerical estimates of the glutamate transporter surface density in different astrocytic sub-cellular compartments. To the best of our knowledge, the hypothesis that the identity of the sub-cellular astrocyte compartment in contact with a synapse is a critical determinant of glutamate escape has not been previously reported. We believe these findings will make a significant impact in our understanding of how astrocytes control glutamate spillover across synapses. A point-to-point reply to the reviewers' concerns is included below.

Thank you for your time and consideration,
Anca Rădulescu and Annalisa Scimemi

Reviewer #1

In this manuscript, Radulescu et al. estimate the surface density of glutamate transporters at the plasma membrane of astrocytes, highlighting differences depending on the sub-cellular compartment, species and mouse age. To do so, they performed dot blotting on protein extracts from mouse hippocampus as well as calculations. Stochastic reaction-diffusion simulations of glutamate uptake in a cube world containing 7 adjacent synapses predict the effect of the local amount of glutamate transporters on the activation of post-synaptic glutamate receptors. Further, 2-photon excitation laser scanning fluorescence microscopy on brain slices treated with protein retention expansion microscopy protocol is used to characterize astrocyte morphology, allowing the generation of 3D reconstructions of astrocytes. Based on the resulting morphological analysis, the authors propose a custom-made code that allows the generation of a bank of 3D models of astrocytes that can be used for in silico simulations and for morphological analysis. Finally, calculations were performed to investigate the surface area that can be occupied by glutamate transporters onto the astrocytic membrane depending on local morphology, notably cylinder versus sphere, thus proposing the maximum amount of transporters on each cellular sub-compartment. Those calculations suggest that the nature of the astrocytic compartment contacting synapses affects the local density of glutamate transporters. Since glutamate uptake affects synaptic activity, glutamate spillover, and is altered in brain disorders such as epilepsy, better characterizing the localization of its effectors, glutamate transporters, is crucial.

While the question investigated by this study is of tremendous importance in the field, I have some concerns regarding the relevance of the methods proposed to address it and regarding the novelty of the results obtained. I would also suggest major reorganization of the manuscript.

We would like to thank the reviewer for the thorough evaluation of our manuscript. We have addressed the concerns raised by the reviewer by: *(i)* reorganizing the entire manuscript and by revising particularly the **Introduction** and **Discussion** sections;*(ii)* moving the math-intense part to the **Supplementary Information** section of the paper; *(iii)* clarifying the assumptions made for all models and calculations; *(iv)* revising and assessing the accuracy of our 3D Monte Carlo model to include estimates on perisynaptic receptor activation; *(v)* activating the repositories for data and code access, which were previously set as 'private'. We believe addressing these concerns has given us the opportunity to streamline their presentation and clarify the relevance of our findings.

MAJOR POINTS

1. ***Discussion of the main insights.*** *My first major concern is that, while the manuscript presents an impressive amount of work, the relevance and novelty of the presented results compared to previous works are a bit unclear and not sufficiently discussed. Key recent articles in the field are notably not cited (see details below).*

The revised manuscripts includes a more in-depth discussion of our own results, and of those presented in more recent works. Briefly: *(i)* we provide new estimates of glutamate transporter surface density in astrocytes of the mouse hippocampus, over a wide age range that had not been previously tested within a single work;*(ii)* to the best of our knowledge, this is the first work that analyzes how crowding effects limit the local expression of glutamate transporters in small sub-cellular compartments and at branching points; *(iii)* this work indicates that the identity of the sub-cellular compartment in contact with a synapse, not only the extent of its astrocytic coverage, is a main determinant of glutamate spillover. For this reason, we believe the surface density estimates of glutamate transporter expression in different sub-cellular compartments provided here will provide an important resource for modeling studies of glutamate uptake in astrocytes and for experimental works focused on understanding the dynamics of synaptic transmission at small central synapses and the mechanisms that control inter-synaptic independence. These points are now discussed in more detail in the revised version of this manuscript.

1.a. ***Distribution of glutamate transporters.*** *Recent electron and super-resolution studies have quantified the density of glutamate transporters with high spatial resolution (see e.g the recent review from Groc & Choquet, Science, 2020 for an overview of the techniques, Ciappelloni et al, Cell Calcium 2017 for single nanoparticle tracking in live astrocytes and quantification of transporter surface diffusion and Heller et al, Methods, 2020 for dSTORM nanoscale localization of individual astrocytic glutamate transporters). Further, Schreiner et al, Journal of Comparative Neurology, 2013, quantified the expression profile of GLAST and GLT-1 in the mouse hippocampus at different postnatal ages: P3–P5; P10–P15; P20–P25 and P60–P70, using immunohistochemistry, western blotting, confocal and STED microscopy. They have reported, similarly to this manuscript, that both GLAST and GLT-1 immunoreactivity increased during postnatal development. Schreiner et al. further used confocal and super-resolution microscopy to characterize the subcellular localization of glutamate transporters. This highlights that the distribution of glutamate transporters in mouse hippocampus during development has already been addressed by previous studies. Advances in the field provided by the submitted manuscript compared to the above-mentioned studies should be clarified.*

The reviewer rightly notes that other works, in addition to the one cited in the original version of our manuscript [2], indicate that there is a non-uniform distribution of GLAST/GLT-1 along the astrocyte membrane. We have clarified this in the **Introduction** section of the paper. Briefly, the existence of this uneven distribution of transporters along the astrocyte membrane was originally obtained using immunolabeling light microscopy [3]. Since then, it has been confirmed using immunolabeling electron and confocal microscopy in acute and organotypic hippocampal slice cultures [4, 5, 6, 7], and more recently using super-resolution STORM and STED multi-color microscopy imaging [8, 9]. These works are cited in the revised manuscript. The work of [9] analyzed the developmental regulation of GLAST/GLT-1 expression at P3–P5; P10–P15; P20–P25 and P60–P70, as noted by the reviewer. According to the authors, the expression of GLAST at P60-70 is similar to that measured at P20-25, whereas that of GLT-1 continues to increase until

P60-70. We analyze a different age range, which extends beyond the one analyzed by [9] (i.e. 2-3 weeks to 21 months) and show that the expression of GLAST/GLT-1 continues to increase until 6 months of age (see **Fig. 3**). None of the previous works provided an estimate of the surface density of GLAST/GLT-1 in different sub-cellular compartments, which is one of the main findings of our work. We would like to thank the reviewer for pointing out works which quantified the surface diffusion coefficient of GLT-1, which has now been incorporated our 3D Monte Carlo models. We have clarified the novelty of these findings and the changes made to the models in the revised manuscript. Note that the references to [10, 11] were omitted because they are reviews of previous works. Instead, when possible, we preferred to included references to the original works.

1.b. ***Assumptions made for the calculations presented in this manuscript need to be discussed.*** *For example, spatial constraints dictating trimer distribution onto the surface of each astrocytic sub-compartment should strongly vary with branching angle.*

The reviewer is correct in that the crowding effect on transporter trimers near a branching point (and hence the overall transporter number and occupancy in a given sub-cellular compartment) depends strongly on the geometry at the branching point. This geometry is rather complex, and depends on the branching angle formed between the daughter and parent branch, and on their diameter. This is now discussed in the revised manuscript. Briefly, since in our model (based on empirical evidence), the branching angles become larger and branches become thinner at increasing branch levels, the geometrical constraints on the trimer distribution differ between branch levels, and between compartments. While the simple modeling assumptions that allow us to populate transporter trimers in cylindrical branch segments work well away from branching points, additional details are necessary to describe the spatial distribution of transporters at branching points, both in terms of the branching point geometry and in terms of constrains on the spatial distribution of these molecules. For example, the membrane surface around a branching point (where the parent branch generates a daughter branch at an angle θ, and after which it continues with decreased diameter) has the shape of a skew truncated cone, with an elliptical hole marking the insertion of the daughter branch onto the parent branch (see blue shaded area in **Fig. S5A**). Because the surface area of the skew truncated cone cannot be expressed as an elementary function of the top and bottom cross-section diameters and height, we approximated its shape to that of a cylinder, with a base radius matching that of the smaller cross-section of the skew truncated cone. This approximation accounts for the loss of a portion of the surface area of the truncated cone at the insertion of the daughter branch, and for the presence of a portion of the surface area of the parent branch that extends beyond the one defined by our cylindrical approximation. We then used further geometric computations to estimate, based on local measures (of diameters and angle) which portions on the surface of the parent and daughter branches cannot be occupied by transporter trimers, due to crowding effects. These approximations are used in our simulation codes, and are now explained in detail in the **Supplementary Information**, together with other technical computations. The new panel A in **Fig. S5** now also illustrates geometrically these assumptions.

1.c. ***There is increasing evidence that EAATs are expressed by other cells than astrocytes (Suarez-Pozos et al, 2020, Neurochem Res) and GLAST can be located on the membrane of the endoplasmic reticulum.*** *This should be discussed. The study compares the density of glutamate transporters in mouse VS rat by comparing their experimental results measured in mice with a study on rats that is more than 20 years old. The results could differ because of a difference in the protocol/laboratory doing the experiment. To assess the difference between mouse and rat glutamate transporters expression, experiments within the same lab on protein extracts from both rat and mouse hippocampus is required.*

The reviewer is correct, in that the expression of the glutamate transporters GLAST/GLT-1 is not limited to astrocytes, but can also be detected in neurons of the hippocampus [12, 13], somatosensory cortex [14] and striatum [15]. However, GLT-1 in neurons represents only $5-10\%$ of its total expression [16]. Perhaps because of this pronounced difference in the expression of these transporters in astrocytic versus neuronal membranes, in previous work analyzing the ultrastructural distributions of GLAST/GLT-1 in the hippocampus, virtually all the immunoreactivity appeared to be related to the astrocytic plasma membranes [5]. For this reason, like [1], we assumed that all the GLAST/GLT-1 molecules isolated in our dot blot experiments

belonged to astrocytes. The possibility that this could introduce a marginal error in our estimates is now discussed in the **Introduction** and **Discussion** sections of the manuscript. The work cited by the reviewer provides a comprehensive list of recent publications showing that GLAST/GLT-1 are also expressed in oligodendrocytes [17]. However, this occurs in preparations other than the mouse hippocampus (e.g. oligodendrocyte cultures, bovine brain sections, rat optic nerve sections, human brain and spinal cord, mouse spinal cord and corpus callosum). To the best of our knowledge, there is currently no evidence of GLAST/GLT-1 expression in oligodendrocytes of the rodent hippocampus. Since this is the brain region that is the focus of the analysis included in this manuscript, further adjustments due to a potential expression of GLAST/GLT-1 in these cells were not made. This allowed us to draw a closer comparison with the results of [1] and in both works the error do to this assumption is thought to be marginal. Furthermore, the estimates of glutamate transporter surface density in rat hippocampal astrocytes obtained originally by [1], are consistent with glutamate transporter density measures estimated electrophysiologically by a number of investigators, which would not happen if there was a significant expression of GLAST/GLT-1 in hippocampal oligodendrocytes [18, 19, 20]. This is now discussed in the revised manuscript. The estimates of density of astrocytic membranes in the rat hippocampus has already been validated by others [13]. The reviewer, howeverm raises a good point in asking us to validate our measures by calculating the surface density of expression of glutamate transporters in the rat hippocampus. To address the reviewer's concern, we repeated the dot blot analysis in hippocampal tissue samples from rats aged 7-8 weeks. The results of this analysis were consistent with those of [1] (**Fig. 3**). This suggests that our own measured of the surface density of GLAST/GLT-1 are unlikely to be biased by major procedural errors. We have broadened the age range of mice used for our analysis (see **Results** section), and their discussion has been changed accordingly (see **Discussion**).

1.d. ***Effect of the density of glutamate transporters on glutamate concentration in the cleft.*** *The model predicts that the density of transporters does not impact the glutamate concentration profile in the synaptic cleft (Fig. 3C). This result is quite unexpected and needs to be discussed and compared with results from other models of tripartite synapses and from experimental data.*

As noted by the reviewer, changing the astrocyte glutamate transporter surface density does not alter the concentration of glutamate in the synaptic cleft. This was analyzed quantitatively in one of our previous publications [21] and by others [22], and is ultimately due to the fact that these transporters are located outside the synaptic cleft. Inside the cleft, passive diffusion is the main mechanism responsible for glutamate clearance. By contrast, astrocyte transporters are responsible for limiting spillover responses away from the release site. This is consistent with a number of quantitative modeling studies, indicating that the result is independent on the use of a specific modeling environment [23, 24, 25, 26, 27, 28]. Although we referred to previous works in the first draft of the manuscript, we have now expanded the discussion of this finding in the **Results** section of the paper.

1.e. ***Spatial resolution of the morphologies used in the study.*** *The model's assumption that the morphology of astrocytes is tree-like is now strongly questioned in the field, with most recent reports highlighting a reticular/spongiform morphology of fine processes: see e.g Shigetomi et al., 2013, . J. Gen. Physiol.; Rusakov, 2015, Nat rev Neurosci and Arizono et al., 2020, Nature Communications. If I understood correctly, the spatial resolution of the reconstruction used to validate the modelled astrocyte morphologies is 0.51 µm, which does not allow to resolve most of astrocytic surface area (75% according to Bindocci et al, Science, 2018). The morphologies presented in this study thus do not account for the fine astrocytic processes of the spongiform domain reported by electron microscopy and super-resolution microscopy. This, as well as the relevance of the proposed geometries, should be critically discussed in the manuscript. Previous morphological analysis of astrocyte morphology should be cited and discussed: Medvedev et al, Phil. Trans. R. Soc. B, 2014; Savtchenko et al, Nature Communications, 2018 and Minge et al, 2021, Frontiers in Cellular Neuroscience. The latter characterized the heterogeneity of astrocyte morphology in the hippocampus during development and is thus crucial to be cited and discussed here.. In any case, the conclusion at the end of the Introduction section "astrocyte tips [. . .] represent a small proportion of the entire astrocytic membrane" and the prediction that most astrocyte surface is contacted by other compartments*

than fine processes is against recent reports in the field.

The reviewer raises a valid point, and rightly notices that even when using proExM and 2P-LSM, our effective spatial resolution does not allow us to capture the fine spongiform processes that emanate from the entire cell membrane of astrocytes. However, these processes can be detected using 3D axial STEM tomography. To overcome the limitation of our original model, we implemented a new one along the lines of the one used by [29]. Small processes, which we called leaves (in the spirit of the reference), were added to all astrocyte branches. Each leaf was modeled as a cylinder of 2 μm length, emerging perpendicularly to the parent branch and randomly distributed along its length. Leaf diameters were generated using the 3/2 rule, weighted so that leaf diameters would range between values of small astrocytic processes detected with our 3D axial STEM tomography analysis. The linear density of the leaves (i.e., their number for a given unit of length of an astrocyte branch) was adjusted to match our data showing that the density of these processes is ~ 4 μm^{-3}. Note that the addition of leaves increased the overall astrocyte surface area by $\sim 1.6\%$. Bindocci *et al.* reported that $\sim 75\%$ of the astrocyte volume lies in the gliapil (i.e., the non core regions, which in their paper include the soma, stem processes with appendages $\geqslant 1$ μm, endfeet). In our manuscript the compartments analogous to these non core regions include both shafts and tips/leaves. The sum of their volumes is $\sim 64\%$ of the entire astrocyte volume. Therefore, our estimates are in agreement with those reported by [30]. The tissue volume fraction occupied by fine astrocyte protrusions, calculated as described in the **Supplementary Information**, was $6.7 \pm 3.5\%$ (n=10), which is consistent with [31] (i.e., $4.0 - 8.9\%$. Therefore, our *in silico* model astrocytes capture the main morphological features of real astrocytes, and their structure is in general agreement with that reported by others. This is now clarified in the text of the revised manuscript.We still find, however, that the majority of the transporters is in the shafts. We explain in the text that this is likely due to crowding effects in small compartments like tips/leaves.

2. ***Manuscript organization.*** *While the text is well written, taken aside few typos such as "this findings", the manuscript lacks conciseness and a logical framework. Conclusions vary between the abstract/introduction/"main points"/conclusion, so that the main insights from the paper are unclear. The manuscript would gain in clarity with a bit of reorganization. Many redundancies are observed, notably between the Methods and Results sections. Citation of figures goes back and forth. To improve the readability of the manuscript, I would suggest to move part of the calculations that are not delivering the main messages of the paper as supporting information (for example Fig. 5-9). The introduction, while showing a deep understanding of the physiology of glutamate transporters, contains non-essential information for this study and could be shortened to improve readability. Most morphological data are presented within tables (e.g Tables 4 and 6), but would be easier to read and interpret if presented as plots.*

We agree with the reviewer that our first submission required some restructuring. To address the reviewer's concerns, we have: *(i)* moved most of the math calculations to the **Supplementary Information** section; *(ii)* included the morphological data that were originally part of **Tables 4,6** in two separate figures (**Fig. 3,5**), to improve readability; *(iii)* revised the **Introduction**; *(iv)* checked possible typos in the text; *(v)* made sure that the citation of figures and tables does not go back and forth; *(vi)* reformatted the sections of the manuscript according to the journal style of PLoS Computational Biology.

3. ***Sensitivity analysis of the model predictions.*** *The model predictions are very interesting. To what extent are they robust depending on parameter values used in the study? For example, as astrocyte-synapse distance is highly variable, to what extent does the glutamate concentration profile predicted by the model depend on this distance?*

This is an interesting point, which has been previously addressed by a number of groups including our own [22, 23, 32, 32, 24, 28, 27]. Briefly, the glutamate concentration profile is filtered at increasing distance from the release site, meaning that it becomes smaller and slower in rise and decay time. This phenomenon can also be appreciated in this manuscript (cf. **Fig. 6E,F**). We have further clarified this point in the **Results** section of the revised manuscript.

In the model, glutamate is released at the centre of the cleft rather than at the membrane of the pre-synaptic element. Does the location of glutamate release impact the model's predictions?

The glutamate concentration is calculated in three regions: the intra-cleft volume over the PSD area and in the annulus surrounding it, and the extracellular volume. Having the release site 10 or 20 nm above the center of the PSD does not change the estimated glutamate concentration in any of these three compartments. Something different, however, could happen if the release site is offset with respect to the center of the cleft (regardless of whether this is 10 or 20 nm above the postsynaptic membrane). We now analyze this additional scenario in **Fig. 7**. We find that glutamate escape from the synaptic cleft is reduced when the release site is located closer to the portion of the synapse contacted by an astrocyte process. Further details are now included as part of the revised manuscript.

The open probability of glutamate receptors are calculated using ChanneLab 2.041213.Does this method provide similar results to 3D Monte Carlo reaction-diffusion simulations that would include those receptors at the post-synaptic membrane?

This method is used because it is more computationally efficient. CellBlender and Channelab use the same Markov models to describe the kinetic properties of receptors and transporters. CellBlender provides a good description of how the probability of occupancy of different transporter states evolves over time (see **Fig. 6C,D**). The surface density of the receptors, however, is 30 times smaller than that of transporters. Because of this, estimating the open probability of receptors from CellBlender can generate noisy results. This limitation can be overcome by running the simulations multiple times, but this comes at a cost of computing time. All our simulations are run 100 times, and the results included in the manuscript represent the average of these many repetitions. Running them 30 times more (i.e. 3,000 seeds) means that each simulation would take at least 30 hours, using our current computational power. Channelab overcomes this limitation, and for this reason, we and others have used this approach extensively to obtain accurate results while also maximizing the efficiency of our computing times [27, 28, 33, 34]. To address this concern, we added a more detailed description of this rationale in the **Methods** section of the paper.

4. ***Code availability.*** *While the code for generating in silico astrocytes is on Github, it seems that the access is restricted so that I could not review or test the code. The availability of the code generating 3D Monte Carlo reaction-diffusion simulations was not mentioned.*

The reviewer is correct. Although all codes (MATLAB and CellBlender) were uploaded on GitHub, the access settings was set to 'private' and for this reason the reviewer could not access it. We now confirm that the repository is publicly available on Github (https://github.com/scimemia/Glutamate-transporters-estimates).

MINOR POINTS

- Numbered lines would be greatly appreciated to facilitate review and comments.

  We added line numbers to the revised version of the manuscript.

- The fact that transporters account for 11-27% of all membrane proteins is surprising and requires discussion.

  This is now discussed in the **Results** section of the manuscript.

- Summary figure: some legend is lacking (spillover/synapse). It could also be interesting to add the effect of transporter density on the extrasynaptic receptor activation as it is highlighted both in the abstract and in the "main points" section.

  We checked the accuracy of all figure legends in the revised version of the manuscript. We ran new simulations to examine the effect of varying glial transporter concentrations on the activation of AMPA and NMDA receptors in the PSD, perisynaptic region and extrasynaptic region (**Fig. 6**).

Reviewer #2

In the project "Estimating the glutamate transporter surface density in mouse hippocampal astrocytes" Radulescu at al., estimate the local surface density of GLAST and GLT-1 in different areas of hippocampal astrocytes in mice aged from 2-3 weeks, 7-8 weeks and 15 months. The authors with experimental data, stochastic model and analytical calculations revealed that the astrocyte ends, which hold the highest density of transporters, represent a small proportion of the entire astrocytic membrane and proposed that most synapses are contacted lower transporter membrane. From my perspective, the manuscript is a good mixture of experimental and simulation data to discover an essential parameter of extracellular glutamate concentration, namely, the spatial distribution of glutamate transporters in mouse hippocampus.

In principle, the work is essential, necessary, with exciting results. However, a significant drawback is that the manuscript is poorly organized. I got the impression that the material was collected in a hurry, leaving something unnecessary and missing something important. The manuscript consists of three practically non-overlapping parts, an experiment to estimate the density of Glu transporters, stochastic modelling of the glutamate dynamics, and an analytical estimate of the maximum density of triangles on various astrocytic surfaces. These parts don't complement each other. It is necessary to reorganize the material to clarify how these three parts complement each other.

Formally, the main idea of the manuscript can be described in the following sentence. The density of glutamate transporters in the mouse hippocampus is higher than in the rat hippocampus, and transporters are mainly not at the tips of the astrocyte (because there are few of them) but on large branches. By the way, the main conclusion that I saw in stochastic modelling is that the higher the density of transporters, the lower the concentration of glutamate. However, this is obvious even without modelling. Thus, the reading of the manuscript raises the question of why, in principle, a model of glutamate diffusion and uptake is needed to define the density of transporters estimated from experiment and surface density calculations. Therefore, at the very beginning, in the introduction, the authors should emphasize what role of stochastic simulations of glutamate plays in determining the density and distribution of glutamate transporters.

However, for the sake of fairness, in general, the results may be of interest to a wide range of readers working in the field of neuron and astrocytes communications. Here the questions and advice to the authors to improve the manuscript.

1. *I did not find any software at this address, and even the page itself is missing.*

We apologize for this. Although the codes were uploaded to our GitHub page, the access setting was set to 'private' and for this reason the reviewer could not access it. The have revised this, so the codes is now publicly available on Github (https://github.com/scimemia/Glutamate-transporters-estimates)

2. *Please use the math format for numbers (10^2 and so on), not a digital format like 1e2 or 1e2.*

All digital formats have been converted to math format, as requested by the reviewer.

3. *Page.10. The authors determined the diffusion region with absorbing boundaries. However, a computation area with a fixed size is required only for a given diffusion mesh. Otherwise, if each particle is independent and has a different diffusion's step, which is very important for complex geometric regions, it makes no sense to determine the size of the calculation's area. It's just that when analyzing diffusion, you can ignore particles that have moved away from the point of release at a certain distance. In this case, there will be no problems with strange absorbing borders. The condition of absorbing boundaries creates an additional flux of these particles, which sharply decreases the concentration of ions inside the diffusion region.*

We would like to clarify that in these simulations, all glutamate molecules have the same diffusion coefficient ($D^* = 3.30 \cdot 10^{-6}$ cm^2/s within the synaptic cleft [35], and $D^* = 1.29 \cdot 10^{-6}$ cm^2/s outside the synaptic cleft to

account for differences in tortuosity). At each time interval, the spatial displacement of each each molecule is the same but its direction differs and obeys Fick's laws of diffusion [36, 37, 38]. We understand the reviewer's concern and agree with it. We repeated our simulations using transparent walls for the world. This is now clarified in the methods section of the paper.

4. *Page. 10. "Astrocyte branch diameter generation." The authors introduce an algorithm for generating a geometric model of astrocytes in silico. In the completed model, the average astrocyte diameter is 1 μm, which means that the surface-to-volume ratio will be 2μm^{-1}. Typically, however, this value is in the order of 20μm^{-1}, which corresponds to a diameter of 100 nm. Some comments are needed here.*

In the cerebellum, the average surface to volume ratio of Bergmann glial cells obtained using EM measures has been given as 13.0 μm^{-1} [39, 40] and 26.2 μm^{-1} in the molecular layer of the rat dentate gyrus [41], and 3.3 μm^{-1} in the cat cortex [42]. In our experiments, the surface to volume ratio of the whole cell was 2.4-2.6 μm^{-1} (see **Fig. 4**). These values are consistent with those reported in previous work by [43], using confocal morphometry. The discrepancy of these measures and those obtained previously with EM has been attributed to cell swelling during the fixation procedure in EM measurements, and to differences in image processing and resolution between confocal microscopy and EM [43]. This is now explained in the **Results** section of the revised manuscript.

5. *The section "Three-dimensional modelling of the Monte Carlo diffusion-reaction" requires a more accurate description of the algorithm for the numerical interaction of glutamate molecules with transporters. Interaction is a critical point in modelling. As far as I understand, the transporters were presented in concentrations and not in individual particles? However, glutamate is represented by individual particles, which still need to be converted into concentration. It is not clear from the method how the average concentration of glutamate was calculated, over what volumes were averaged, etc. Moreover, it is necessary to say a few words why such a model and such parameters of transporters kinetics were chosen.*

In this model, the transporters are represented as individual molecules with a given surface density. CellBlender calculates the surface area of the region over which the transporters need to be distributed. Based on their surface density values, it calculates how many transporters need to be scattered within the region of interest. Each transporter is a molecule capable of interacting with a diffusing glutamate molecule. Therefore, in our simulations, the transporters are not presented with a given concentration of glutamate. This is clarified in the **Methods** section of the paper. The rationale for the selection of the kinetic scheme for transporters and a more thorough description on how the glutamate concentration estimates were derived is also included in the **Methods**.

6. *The part "Glutamate transporter spatial distribution" is interesting. The problem of the optimal arrangement of figures on the surface is complex and requires profound investigation. Furthermore, this is, of course, an essential part of the manuscript. However, proteins are incorporated into membranes randomly, so the number of randomly included particles (non-overlapping) will always be much smaller than the maximum quantity. Maybe the authors will find a method to estimate the deviation of the number of transporters at random distribution from their maximum number.*

We use the optimal arrangement of transporters on the astrocyte cell membrane as a way to determine the maximum number of transporters that can be placed along the astrocyte plasma membrane. This organization is useful for computational purposes, but of course does not represent a realistic scenario as glutamate transporters are not the only molecules expressed on the plasma membrane of these cells. Their distribution within each sub-cellular compartment, for simplicity, is thought to be random. Estimating and understanding the deviation from the theoretical maximum in different compartments is precisely one of the focus points of our paper, as pointed out by the reviewer, and is reported in **Fig. 5G,H**. Here, we calculate: *(i)* the ratio between the actual density of transporters in each compartment and the maximal one estimated through the model (**Fig. 5G**); *(ii)* the ratio between the effective surface area of the astrocyte plasma membrane occupied by the transporters and the maximal one they could occupy in different subcellular compartments, once crowding effects are taken into account (**Fig. 5H**). This is now explained in

detail in the revised version of the manuscript. Note that our calculations are now more complex than in the first version of this manuscript, as they now include detailed descriptions of how the transporter density was calculated at branching points and in astrocytes with more complex morphology than we had before (they now include leaves, which were not present in the first submission of this manuscript).

7. *Fig.1D,E. Surface density in um-2 (not um-3).*

The reviewer is correct. The y-axis label has been corrected accordingly.

8. *Page. 20 "An increase in astrocytic glutamate transporter expression did not lead to changes in the glutamate concentration profile in the synaptic cleft (**Fig. 3C**)". It is necessary to correct this phrase since the glutamate dynamic is shown at the synaptic cleft centre, and this is why the concentration-independent of transporters. However, at the cleft edge, the concentration can vary depending on the density of transporters. In any case, that is related to my previous question, how do the authors calculate the glutamate concentration inside the cleft and outside?*

We addressed this concern in the revised version of the manuscript. We now estimate the concentration of glutamate in the cleft volume above the PSD and in the surrounding perisynaptic region (i.e. the outer annulus of the synaptic cleft). The extracellular glutamate concentration is calculated based on the number of glutamate molecules in the world, which is the extracellular volume contained in the world of the simulation. This is now explained in the text and the results of these simulations are now included in the revised version of **Fig. 6**.

Reviewer #3

The study investigates the effects of varied expression of glutamate transporters on astrocytes of the mouse hippocampus, on synaptic signaling. This an important study for various reasons. The activity of transporters is critical for the specificity of signaling and preventing excitotoxicity. Consequently, several neurological disorders can be correlated with the aberrational activity of transporters. Existing data is based on studies on adult rats, however, most experiments and models use mice in various stages of development and continue to use data based on rat experiments. This study extensively reports on expression levels of transporters across age groups and localizations of transporter subtypes in different parts of the cell in a mouse using a wide variety of experimental techniques. The study then uses this data to develop physiologically realistic 3d models of the astrocytic processes and accounts for varying coverage of synapses in the hippocampus. The results quantitatively describe how changes in expression of transporters modify synaptic signaling via activation of extrasynaptic AMPA and NMDA receptors. It was a pleasure to read the manuscript. The results are important for several future studies on synaptic signaling in the hippocampus. The methods are described with great care and detail. This manuscript is an example of how detailed biophysical modeling can provide physiological insights that can chart the course of future investigations.

We would like to thank this reviewer for the positive feedback on our work. We agree that the data presented here will greatly benefit the modeling community, by providing estimates that until today were based on a rodent species (rats), whether the surface density of glutamate transporters is much smaller than in mice. Given that there is a significant proportion of experimental work done in mice, it is important that these estimates are used in modeling studies to further improve the accuracy with which they can reproduce the functional properties of living synapses.

References

[1] Knut P Lehre and Niels C Danbolt. The number of glutamate transporter subtype molecules at glutamatergic synapses: chemical and stereological quantification in young adult rat brain. *The Journal of Neuroscience*, 18(21):8751–8757, 1998.

[2] Mariko Kato Hayashi and Masato Yasui. The transmembrane transporter domain of glutamate transporters is a process tip localizer. *Scientific Reports*, 5(1):1–8, 2015.

[3] NC Danbolt, J Storm-Mathisen, and BI Kanner. A $\mathrm{Na}^{+}/\mathrm{K}^{+}$ coupled-glutamate transporter purified from rat brain is located in glial cell processes. *Neuroscience*, 51(2):295–310, 1992.

[4] Jeffrey D Rothstein, Lee Martin, Allan I Levey, Margaret Dykes-Hoberg, Lin Jin, David Wu, Norman Nash, and Ralph W Kuncl. Localization of neuronal and glial glutamate transporters. *Neuron*, 13(3):713–725, 1994.

[5] Farrukh A Chaudhry, Knut P Lehre, Menno van Lookeren Campagne, Ole P Ottersen, Niels C Danbolt, and Jon Storm-Mathisen. Glutamate transporters in glial plasma membranes: highly differentiated localizations revealed by quantitative ultrastructural immunocytochemistry. *Neuron*, 15(3):711–720, 1995.

[6] Andrea Minelli, P Barbaresi, RJ Reimer, RH Edwards, and F Conti. The glial glutamate transporter glt-1 is localized both in the vicinity of and at distance from axon terminals in the rat cerebral cortex. *Neuroscience*, 108(1):51–59, 2001.

[7] Adrienne M Benediktsson, Glen S Marrs, Jian Cheng Tu, Paul F Worley, Jeffrey D Rothstein, Dwight E Bergles, and Michael E Dailey. Neuronal activity regulates glutamate transporter dynamics in developing astrocytes. *Glia*, 60(2):175–188, 2012.

[8] Janosch Peter Heller, Tuamoru Odii, Kaiyu Zheng, and Dmitri A Rusakov. Imaging tripartite synapses using super-resolution microscopy. *Methods*, 174:81–90, 2020.

[9] Alexandra E Schreiner, Simone Durry, Tomomi Aida, Martin C Stock, Ulrich Rüther, Kohichi Tanaka, Christine R Rose, and Karl W Kafitz. Laminar and subcellular heterogeneity of glast and glt-1 immunoreactivity in the developing postnatal mouse hippocampus. *Journal of Comparative Neurology*, 522(1):204–224, 2014.

[10] Laurent Groc and Daniel Choquet. Linking glutamate receptor movements and synapse function. *Science*, 368(6496), 2020.

[11] Silvia Ciappelloni, Ciaran Murphy-Royal, Julien P Dupuis, Stéphane HR Oliet, and Laurent Groc. Dynamics of surface neurotransmitter receptors and transporters in glial cells: single molecule insights. *Cell Calcium*, 67:46–52, 2017.

[12] Weizhi Chen, Veeravan Mahadomrongkul, Urs V Berger, Merav Bassan, Tara DeSilva, Kohichi Tanaka, Nina Irwin, Chiye Aoki, and Paul A Rosenberg. The glutamate transporter glt1a is expressed in excitatory axon terminals of mature hippocampal neurons. *Journal of Neuroscience*, 24(5):1136–1148, 2004.

[13] DN Furness, Y Dehnes, AQ Akhtar, DJ Rossi, M Hamann, NJ Grutle, V Gundersen, S Holmseth, KP Lehre, K Ullensvang, M Wojewodzica, Y Zhou, D Attwell, and NC Danbolt. A quantitative assessment of glutamate uptake into hippocampal synaptic terminals and astrocytes: new insights into a neuronal role for excitatory amino acid transporter 2 (eaat2). *Neuroscience*, 157(1):80–94, 2008.

[14] Marcello Melone, Michele Bellesi, and Fiorenzo Conti. Synaptic localization of glt-1a in the rat somatic sensory cortex. *Glia*, 57(1):108–117, 2009.

[15] Geraldine T Petr, Laurel A Schultheis, Kayla C Hussey, Yan Sun, Janet M Dubinsky, Chiye Aoki, and Paul A Rosenberg. Decreased expression of glt-1 in the r6/2 model of huntington's disease does not worsen disease progression. *European Journal of Neuroscience*, 38(3):2477–2490, 2013.

[16] Theresa S Rimmele and Paul A Rosenberg. Glt-1: the elusive presynaptic glutamate transporter. *Neurochemistry International*, 98:19–28, 2016.

[17] Edna Suárez-Pozos, Elizabeth J Thomason, and Babette Fuss. Glutamate transporters: expression and function in oligodendrocytes. *Neurochemical Research*, 45(3):551–560, 2020.

[18] Dwight E Bergles and Craig E Jahr. Synaptic activation of glutamate transporters in hippocampal astrocytes. *Neuron*, 19(6):1297–1308, 1997.

[19] Michiko Takahashi, Monique Sarantis, and David Attwell. Postsynaptic glutamate uptake in rat cerebellar Purkinje cells. *The Journal of Physiology*, 497(2):523–530, 1996.

[20] Thomas S Otis and Michael P Kavanaugh. Isolation of current components and partial reaction cycles in the glial glutamate transporter eaat2. *The Journal of Neuroscience*, 20(8):2749–2757, 2000.

[21] Kaiyu Zheng, Annalisa Scimemi, and Dmitri A Rusakov. Receptor actions of synaptically released glutamate: the role of transporters on the scale from nanometers to microns. *Biophysical Journal*, 95(10):4584–4596, 2008.

[22] Dmitri A Rusakov and Dimitri M Kullmann. Extrasynaptic glutamate diffusion in the hippocampus: ultrastructural constraints, uptake, and receptor activation. *The Journal of Neuroscience*, 18(9):3158–3170, 1998.

[23] Boris Barbour. An evaluation of synapse independence. *The Journal of Neuroscience*, 21(20):7969–7984, 2001.

[24] Nikolay Gavrilov, Inna Golyagina, Alexey Brazhe, Annalisa Scimemi, Vadim Turlapov, and Alexey Semyanov. Astrocytic coverage of dendritic spines, dendritic shafts, and axonal boutons in hippocampal neuropil. *Frontiers in Cellular Neuroscience*, 12:248, 2018.

[25] Jeffrey S Diamond and Craig E Jahr. Transporters buffer synaptically released glutamate on a submillisecond time scale. *The Journal of Neuroscience*, 17(12):4672–4687, 1997.

[26] Leonid P Savtchenko and Dmitri A Rusakov. Extracellular diffusivity determines contribution of high-versus low-affinity receptors to neural signaling. *Neuroimage*, 25(1):101–111, 2005.

[27] Annalisa Scimemi and Marco Beato. Determining the neurotransmitter concentration profile at active synapses. *Molecular Neurobiology*, 40(3):289–306, 2009.

[28] Annalisa Scimemi, Hua Tian, and Jeffrey S Diamond. Neuronal transporters regulate glutamate clearance, NMDA receptor activation, and synaptic plasticity in the hippocampus. *The Journal of Neuroscience*, 29(46):14581–14595, 2009.

[29] Leonid P Savtchenko, Lucie Bard, Thomas P Jensen, James P Reynolds, Igor Kraev, Nikolay Medvedev, Michael G Stewart, Christian Henneberger, and Dmitri A Rusakov. Disentangling astroglial physiology with a realistic cell model in silico. *Nature Communications*, 9(1):1–15, 2018.

[30] Erika Bindocci, Iaroslav Savtchouk, Nicolas Liaudet, Denise Becker, Giovanni Carriero, and Andrea Volterra. Three-dimensional ca2+ imaging advances understanding of astrocyte biology. *Science*, 356(6339), 2017.

[31] Nikolai Medvedev, Victor Popov, Christian Henneberger, Igor Kraev, Dmitri A Rusakov, and Michael G Stewart. Glia selectively approach synapses on thin dendritic spines. *Philosophical Transactions of the Royal Society B: Biological Sciences*, 369(1654):20140047, 2014.

[32] Jeffrey S Diamond. Deriving the glutamate clearance time course from transporter currents in CA1 hippocampal astrocytes: transmitter uptake gets faster during development. *The Journal of Neuroscience*, 25(11):2906–2916, 2005.

[33] Amanda M Sweeney, Kelsey E Fleming, John P McCauley, Marvin F Rodriguez, Elliot T Martin, Alioscka A Sousa, Richard D Leapman, and Annalisa Scimemi. PAR1 activation induces rapid changes in glutamate uptake and astrocyte morphology. *Scientific Reports*, 7:43606, 2017.

[34] Jeffrey S Diamond. Neuronal glutamate transporters limit activation of NMDA receptors by neurotransmitter spillover on CA1 pyramidal cells. *The Journal of Neuroscience*, 21(21):8328–8338, 2001.

[35] Thomas A Nielsen, David A DiGregorio, and R Angus Silver. Modulation of glutamate mobility reveals the mechanism underlying slow-rising ampar epscs and the diffusion coefficient in the synaptic cleft. *Neuron*, 42(5):757–771, 2004.

[36] Joel R Stiles, Dirk Van Helden, Thomas M Bartol, Edwin E Salpeter, and Miriam M Salpeter. Miniature endplate current rise times less than 100 microseconds from improved dual recordings can be modeled with passive acetylcholine diffusion from a synaptic vesicle. *Proceedings of the National Academy of Sciences*, 93(12):5747–5752, 1996.

[37] Joel R Stiles, Thomas M Bartol, et al. Monte carlo methods for simulating realistic synaptic microphysiology using mcell. *Computational Neuroscience: Realistic Modeling for Experimentalists*, pages 87–127, 2001.

[38] Rex A Kerr, Thomas M Bartol, Boris Kaminsky, Markus Dittrich, Jen-Chien Jack Chang, Scott B Baden, Terrence J Sejnowski, and Joel R Stiles. Fast monte carlo simulation methods for biological reaction-diffusion systems in solution and on surfaces. *SIAM Journal on Scientific Computing*, 30(6):3126–3149, 2008.

[39] Jens Grosche, Vitali Matyash, Thomas Möller, Alexej Verkhratsky, Andreas Reichenbach, and Helmut Kettenmann. Microdomains for neuron–glia interaction: parallel fiber signaling to bergmann glial cells. *Nature Neuroscience*, 2(2):139–143, 1999.

[40] Jens Grosche, Helmut Kettenmann, and Andreas Reichenbach. Bergmann glial cells form distinct morphological structures to interact with cerebellar neurons. *The Journal of Neuroscience Research*, 68(2):138–149, 2002.

[41] Kiyoshi Hama, Tatsuo Arii, Eisaku Katayama, Maryann Marton, and Mark H Ellisman. Tri-dimensional morphometric analysis of astrocytic processes with high voltage electron microscopy of thick golgi preparations. *The Journal of Neurocytology*, 33(3):277–285, 2004.

[42] V Williams, RG Grossman, and Sharon M Edmunds. Volume and surface area estimates of astrocytes in the sensorimotor cortex of the cat. *Neuroscience*, 5(7):1151–1159, 1980.

[43] Alexandr Chvátal, Miroslava Anděrová, and Frank Kirchhoff. Three-dimensional confocal morphometry–a new approach for studying dynamic changes in cell morphology in brain slices. *Journal of Anatomy*, 210(6):671–683, 2007.
