# Supplementary material for: Estimating the glutamate transporter surface density in distinct sub-cellular compartments of mouse hippocampal astrocytes
Source: PLoS Comput Biol. 2022 Feb 4;18(2):e1009845. doi: 10.1371/journal.pcbi.1009845 (PMC8849624; doi:10.1371/journal.pcbi.1009845)
Supplement: Attachment — Submitted filename: Plos_Comp_Bio_rev2_letter.pdf [file pcbi.1009845.s009.pdf]

Dear Dr. Berry,

Thank you again for reviewing our manuscript. The additional comments raised by Reviewer 1 have now been addressed. A point-to-point reply to the reviewers' concerns is included below.

Thank you for your time and consideration,

Anca Rădulescu and Annalisa Scimemi

Reviewer #1

The authors have addressed my previous comments. A lot of additional work and experiments/simulations have been performed and the manuscript has greatly improved. My previous comments have been carefully read and answered.I however still have some major concerns regarding the manuscript in its current form

We thank the reviewer for appreciating the substantial revisions that were made to the original version of this manuscript and value the careful revisions provided here. We have now addressed all remaining concerns.

Major concerns

My main concern probably resides in the lack of any statistical analysis in the paper. For example, similarity between the results obtained in the study and other works is often asserted without any justification/quantification (e.g considering that 65% is "close enough" to 75%).

Corrected. To address the reviewer's concern, we have now added information on statistical comparisons between experimental and model data in the figure legends. We have also added a specific section on **Statistical analysis**. Since we are unable to perform meaningful statistical comparisons with data collected from other labs and published in previous works, we have revised our statements in the section that the reviewer refers to.

Further, no analysis is presented to quantify the similarity between the properties of the model and the experimental measurements.

Corrected. Statistical significance values are now included in the legend of **Fig. 4**.

Accordingly, there is no Data analysis/Statistical analysis section in the Methods section. Overall, the manuscript would greatly improve by using data analysis techniques, describing and justifying them (see Makin & Orban de Xivry, 2019, eLife, DOI: 10.7554/eLife.48175).

Corrected. A **Statistical analysis** section is now added to the manuscript.

Although all the model and calculation assumptions are now clarified in the text, some of the assumptions are hard to understand. For example, the authors assert that the proportion of GLT-1 expressed by neurons is negligible while stating that they account for 5-10% of its total expression in the brain (which is not negligible). Why not including those 5-10% in the calculations rather than assuming that all GLT-1 is astroglial?

As indicated in the text, we did not include corrections for neuronal GLT-1 because we wanted our results to be consistent with those obtained in previous works in rats [1, 2]. This allows us to make a direct comparison between our results and those previously obtained by others. Our statement in the main text was not meant to dismiss the potential value and physiological relevance of neuronal GLT-1, but rather to point out to the fact that our estimates are not going to be radically different when accounting for the presence of neuronal GLT-1. Due to the multiple approximations that were necessary to obtain the data described in this manuscript, the estimates provided here should be considered ballpark figures for the surface density

of expression of GLT-1. We hope this clarifies our choice. An additional note on the lack of corrections for neuronal GLT-1 is now added to the legend of **Fig. 3.**

I have concerns regarding the quality of the reconstruction of the astrocyte leaves in Figure 2. The resolution of the method should be clearly stated and comparison with shapes/morphologies previously obtained by other groups should be mentioned.

Corrected. The spatial resolution of the axial STEM tomography analysis is only a few nm. This information has now been added to the main text together with supporting references.

Further, if I understood correctly, it seems that any non-neuronal element near the synapse was considered being an astrocyte.

Corrected. Astrocytes were identified based on structural features previously described by [3], which include the presence of intermediate filament bundles in a relatively clear cytoplasm and lack of synaptic vesicles. Other non-neuronal cells were not included in our reconstructions. This is now clarified in the Methods section of the revised manuscript.

Conclusions are often too assertive. For example, the results from this study suggest but do not show (l.39) that there are stark differences in transporter density depending on the subcellular compartment.

Corrected. We revised and toned down some of the conclusions, as suggested by the reviewer.

Another example: the authors state that the density of the transporters decrease with age while, in Fig5, the decrease of surface density at 15 months is followed by an increase at 21 months old. This latter is however surprising and would be interesting to discuss.

Corrected. The increase in glutamate transporter expression that the reviewer points to (i.e., between 15 months and 21 months) is not statistically significant ($p=0.22$). This information is now added to the revised manuscript. In view of this, we do not feel there is a strong scientific motivation to discuss this further in the manuscript.

Finally, the speculations regarding the 3/2 rule applicable to astrocytes (l599-603) are way too assertive compared to the degree to which it was proven in the paper.

Corrected. We toned down this conclusion.

Some of the results are surprising and require more discussion. For example, l363-366, the authors suggest that at 6 months old, 100% of the astrocyte surface area in leaves would be occupied by transporters. This is however highly unlikely as astrocytes express other proteins in their PAPs (see Mazaré et al, 2020, Cell Reports), including some located at the plasma membrane.

Corrected. This is a valid point, which we discuss in more length in a paragraph added to the **Discussion** section of this manuscript. Briefly, PAPs are different entities compared to tips/leaves, so the fact that there are transcripts for molecules other than GLT-1 in PAPs is not in conflict with our data in astrocytes of 6 month old mice.

The choice of plots to present the data is sometimes misleading, notably in the section presenting the modeling results. As reaction-diffusion simulations are performed, I do not understand why Fig 6F,H and J are presented with lines rather than scatter/bar plots. Error bars are also lacking.

We plotted the results as lines to avoid any potential overlap between symbols that would make visualization of subtle differences more difficult. For this reason, a scatter plot or a bar graph would not be appropriate to display these results. The reviewer is correct that the data represent only the mean values. This is due to the fact that when the number of seeds is large, as is the case in this type of simulations, the error bar becomes so small that it is imperceptible. For this reason, it is a common practice to report only the mean values [4, 5, 6, 7, 8, 9, 10, 11, 12]. The normalized values in panels D, F, H, J are normalized values obtained from the peak ratio of the results shown in panels C, E, G I, so they do not have an error bar. We

have now clarified this in the figure legend.

Importantly, the right panels from Fig8 are misleading and give the impression that simulations were performed with numerous values of astrocyte-release site distance, while only 3 values were tested (close, far, center). Here also, the variability depending on seed value (error bar/STD) should be added to the plots (if the model is indeed a reaction-diffusion model, see also next point).

Corrected. See response to previous point. This is now clarified in the figure legend. A more explicit description of the three different types of simulations described in this figure has been added to the legend.

Information on the model is lacking, such as parameter values (other than geometric parameter values) and the associated references. Presenting them in a schematic as well as a table, for example, is crucial for reproducibility purposes. Further, the details of the modeling technique used are still unclear to me. Indeed, the authors state that they are performing reaction-diffusion simulations while it seems that the model is in fact compartmental (divided into 2 micrometer cube cubes according to Supplemental Fig S6?). Could it be possible to clarify that in the Methods section?

Corrected. There are multiple models in this work. The reaction-diffusion 3D Monte Carlo model described in the **Methods** section is the one presented in **Fig. 6-8**. The corresponding references are all listed in the **Methods** section. Additional information about the geometrical model, first presented in **Fig. 4**, was also presented in the **Methods** section but has now been expanded to address this concern. To clarify, the geometrical model of astrocytes does not involve any compartmental aspect for its geometric construction. In this model, the 2 μm cube partition that the reviewer refers to was only used *post-hoc*, to quantify the volume fraction of the neuropil occupied by the model astrocyte and compare it with previous theoretical and experimental estimates obtained using a similar partitioning approach [13, 14]. This has now been clarified in the manuscript text (lines 292-298), with references to the **Methods** section, as well as in the legend of **Fig. 6** in the **Supplementary Information**.

A lot of the conditions chosen to create the astrocyte morphologies still seem arbitrary (example: the length, orientation, distribution of the leaves).

Corrected. The length (mean 0.7 μm) and orientation of leaves (orthogonal to the parent branch) were chosen to be consistent with those used by Savtchenko et al. (Nature Communications, 2018). In this reference (specifically, in **Fig. 3** of their **Supplementary Information**), the astroglial processes were reconstructed using 60 nm thick serial EM sections and showed that the leaf length varies within the range 0.5-1.25 μm. Therefore, in our model, we chose our leaf length parameter to be in the middle of this range (i.e., 0.7 μm). The linear density of leaves could not be validated with the Savtchenko measures, since we modeled a discrete distribution of leaf protrusions on branches, while this reference modeled them using a 3D bundle approach. We fine-tuned this parameter so that the other (*post-hoc*,dependent) cellular measures (such as branch density per neuropil volume, leaf diameter and Sholl profile) were in close agreement with our own empirical data. This was now clarified in the text, upon introduction of these parameters.

Minor concerns

The reason why the model contains 7 synapses, while glutamate spillover seems to be only monitored at one synapse, is unclear.

Corrected. This is now clarified in the **Methods** section of the manuscript.

The term 'clustering' should be defined and used carefully, as it commonly refers to non-homogeneous distributions of transporters within nanodomains. In this study, the transporters seem to be heterogeneously distributed onto the surface of the astrocyte.

Corrected. We have confirmed that the use of this word is appropriate when included in the text. We have also added a further clarification and verified that the word is used appropriately when citing other works.

Calling the extracellular space outside the cleft "extra-cellular space" is misleading as the cleft itself also consists in extracellular space.

Corrected. This is now clarified in all instances where this notation could lead to ambiguous interpretations about the meaning of this term.

Most of the new paragraphs in the introduction section discuss the choices made during the study and the approximations and calculations (l66-100, 101-109, l111-129). The manuscript would probably gain in clarity if this text was transferred to the Discussion & Supplemental sections.

The sections mentioned by the reviewer provide a summary of experimental approaches used before our study, and that motivated our analysis of glutamate transporter expression in mouse astrocytes at different ages. Without this information, we would lack the scientific motivation that prompted our analysis. For this reason, we would respectfully request these sections to remain in the Introduction.

l151-162 + Fig1 probably belong to the Methods section.

This is an important figure for this manuscript, as it represents the foundation for all our glutamate transporter density measures. We feel that moving it to the **Methods** section would detract attention to its relevance, and would not to justice to the conceptual relevance of the data that is being presented here. For these reasons, we respectfully request to keep this figure and corresponding text in the main section of this manuscript.

Definition of the terminology, notably the definition of shafts vs leaves, is sometimes lacking. This is crucial as those terms can mean different things depending on the laboratory (it would be nice if the community reaches a consensus on this terminology!)

Corrected. This information has now been added to the main text of the manuscript.

Fig7: why testing only 3 data points (close, center, far)? At what time is the probability of occupancy computed? Is it the average from the whole simulation time?

Corrected. We chose these three data points as the ones that could represent the most extreme scenarios, as they cover the whole range of distances that a release site can be: from the closest to the furthest away from the neighboring glial sheet. This is now clarified in the main text of the manuscript. The normalized occupancy values shown in **Fig. 7** were obtained by comparing the peak values for ToG, TiG, Glut, AMPA and NMDA. For To, we compared the trough (i.e. minimum value of To). This is now explained in the legend of **Fig. 7.**

Fig 7 and 8 could probably be combined as they investigate the same question.

Combining these figures would generate a figure with 11 panels. In the interest of clarity, given that the journal allows to have a maximum of eight figures in the main text, we think it is best to keep these figures separate.

How do the proposed morphologies compare to the in silico astrocyte morphologies provided by e.g. Savtchenko et al, Nat. Neuroscience, 2018?

Corrected. This is now clarified in the text, where we now provide further details on how our methods agree and deviate from the Savtchenko model. Briefly, some aspects of our geometry are compatible with Savtchenko et al. 2018. Others, however, are unique because they are driven by our own data, and by our specific aim of understanding cell-compartment differences in the distribution of glutamate transporters.

l460-461: part of the sentence is missing

Corrected. The sentence that the reviewer points out reads: "We do not anticipate species recognition to be an issue for the GLT-1 antibody, however, because the antigen sequence is 100% identical in humans, rats

and mice." We could find any grammatical mistake with it and the sentence reads as complete. To avoid any potential confusion, we rephrased it.

The blender files lack instructions to allow other users to be able to smoothly reuse the code. The required software and repositories to run the code should be clearly listed in the README file.

Corrected. We have edited the README file for the CellBlender simulations in Github to include specifications about the version of CellBlender and Blender used to run these simulations. All other parameters are described in the corresponding Methods section of this paper, which is also included with a link as the reference for the README file on Github. As long as Blender has the CellBlender add-on installed, the user does not need to modify any parameter and everything should run smoothly by clicking the Run button.

References

[1] Knut P Lehre and Niels C Danbolt. The number of glutamate transporter subtype molecules at glutamatergic synapses: chemical and stereological quantification in young adult rat brain. *The Journal of Neuroscience*, 18(21):8751–8757, 1998.

[2] Farrukh A Chaudhry, Knut P Lehre, Menno van Lookeren Campagne, Ole P Ottersen, Niels C Danbolt, and Jon Storm-Mathisen. Glutamate transporters in glial plasma membranes: highly differentiated localizations revealed by quantitative ultrastructural immunocytochemistry. *Neuron*, 15(3):711–720, 1995.

[3] Rachel Ventura and Kristen M Harris. Three-dimensional relationships between hippocampal synapses and astrocytes. *The Journal of Neuroscience*, 19(16):6897–6906, 1999.

[4] Annalisa Scimemi, Hua Tian, and Jeffrey S Diamond. Neuronal transporters regulate glutamate clearance, NMDA receptor activation, and synaptic plasticity in the hippocampus. *The Journal of Neuroscience*, 29(46):14581–14595, 2009.

[5] Annalisa Scimemi and Jeffrey S Diamond. The number and organization of ca2+ channels in the active zone shapes neurotransmitter release from schaffer collateral synapses. *Journal of Neuroscience*, 32(50):18157–18176, 2012.

[6] Boris Barbour. An evaluation of synapse independence. *The Journal of Neuroscience*, 21(20):7969–7984, 2001.

[7] Dmitri A Rusakov and Dimitri M Kullmann. Extrasynaptic glutamate diffusion in the hippocampus: ultrastructural constraints, uptake, and receptor activation. *The Journal of Neuroscience*, 18(9):3158–3170, 1998.

[8] Jeffrey S Diamond. Neuronal glutamate transporters limit activation of NMDA receptors by neurotransmitter spillover on CA1 pyramidal cells. *The Journal of Neuroscience*, 21(21):8328–8338, 2001.

[9] Jeffrey S Diamond. Deriving the glutamate clearance time course from transporter currents in CA1 hippocampal astrocytes: transmitter uptake gets faster during development. *The Journal of Neuroscience*, 25(11):2906–2916, 2005.

[10] Amanda M Sweeney, Kelsey E Fleming, John P McCauley, Marvin F Rodriguez, Elliot T Martin, Alioscka A Sousa, Richard D Leapman, and Annalisa Scimemi. PAR1 activation induces rapid changes in glutamate uptake and astrocyte morphology. *Scientific Reports*, 7:43606, 2017.

[11] Kaiyu Zheng, Annalisa Scimemi, and Dmitri A Rusakov. Receptor actions of synaptically released glutamate: the role of transporters on the scale from nanometers to microns. *Biophysical Journal*, 95(10):4584–4596, 2008.

[12] Leonid P Savtchenko and Dmitri A Rusakov. Extracellular diffusivity determines contribution of high- versus low-affinity receptors to neural signaling. *Neuroimage*, 25(1):101–111, 2005.

[13] Leonid P Savtchenko, Lucie Bard, Thomas P Jensen, James P Reynolds, Igor Kraev, Nikolay Medvedev, Michael G Stewart, Christian Henneberger, and Dmitri A Rusakov. Disentangling astroglial physiology with a realistic cell model in silico. *Nature Communications*, 9(1):1–15, 2018.

[14] Eiji Shigetomi, Eric A Bushong, Martin D Haustein, Xiaoping Tong, Olan Jackson-Weaver, Sebastian Kracun, Ji Xu, Michael V Sofroniew, Mark H Ellisman, and Baljit S Khakh. Imaging calcium microdomains within entire astrocyte territories and endfeet with gcamps expressed using adeno-associated viruses. *Journal of General Physiology*, 141(5):633–647, 2013.
